# Analysis of a household-scale model for the invasion of Wolbachia into a resident mosquito population

**DOI:** 10.1007/s00285-025-02332-8

**Published:** 2025-12-22

**Authors:** Abby Barlow, Sarah Penington, Ben Adams

**Affiliations:** Department of Mathematical Sciences, University of Bath, Claverton Down, Bath, BA2 7AY Somerset United Kingdom

**Keywords:** Wolbachia, Household model, Quasi-stationary distribution, Continuous-time Markov chain, Invasion, Bistability, Primary: 92D40, secondary: 92D30, 92D25, 60J28

## Abstract

In areas infested with Aedes aegypti mosquitoes it may be possible to control dengue, and some other vector-borne diseases, by introducing Wolbachia-infected mosquitoes into the wildtype population. Thus far, empirical and theoretical studies of Wolbachia release have tended to focus on the dynamics at the community scale. However, Ae. aegypti mosquitoes typically dwell in and around the same houses as the people they bite and it can be insightful to explore what happens at the household scale where small population sizes lead to inherently stochastic dynamics. Here we use a continuous-time Markov framework to develop a stochastic household model for small populations of wildtype and Wolbachia-infected mosquitoes. We investigate the transient and long term dynamics of the system, in particular examining the impact of stochasticity on the Wolbachia invasion threshold and bistability between the wildtype-only and Wolbachia-only steady states previously observed in deterministic models. We focus on the influence of key parameters which determine the fitness cost of Wolbachia infection and the probability of Wolbachia vertical transmission. Using Markov and matrix population theory, we derive salient characteristics of the system including the probability of successful Wolbachia invasion, the expected time until invasion and the probability that a Wolbachia-infected population reverts to a wildtype population. These attributes can inform strategies for the release of Wolbachia-infected mosquitoes. In addition, we find that releasing the minimum number of Wolbachia-infected mosquitoes required to displace a resident wildtype population according to the deterministic model, only results in that outcome about 20% of the time in the stochastic model; a significantly larger release is required to reach a steady state composed entirely of Wolbachia-infected mosquitoes 90% of the time.

## Introduction

Dengue is a vector-borne disease with an estimated global burden of 100 to 400 million cases and 40, 000 deaths per year (Zeng et al. 2021). It circulates endemically across tropical and subtropical regions, affecting over 100 countries (Hasan et al. 2016; Bhatt et al. 2013), and local transmission has recently been observed in parts of Europe (WHO 2023). The viral infection is passed to humans via the bite of infected mosquitoes. Its most common vector is the Aedes aegypti mosquito (WHO 2023). Aedes aegypti is predominantly found in urban and sub-urban areas. It favours artificial water containers, such as unsealed cisterns, tyres and empty plant pots as habitats to lay eggs (ECDC 2023) and has benefited from rapid growth and urbanisation of the human population (Trewin et al. 2021).

Dengue prevention methods typically focus on reducing or eliminating the vector population. Spraying insecticides both indoors and outdoors is often used to suppress the mosquitoes. However, complete elimination requires continual spraying, which can lead to the mosquitoes building up resistance to the insecticides. Dengue vaccines have been developed, but currently have limited scope since they are only advised for individuals who have previously contracted the virus (CDC 2024). An alternative dengue control method that has been actively explored in recent years involves infecting the mosquitoes with Wolbachia. Wolbachia are a group of intracellular bacteria found in arthropods and nematodes (Werren et al. 2008; Hughes and Britton 2013). They can alter their host’s biology in numerous ways. When present in mosquitoes such as Aedes aegypti, the bacteria can reduce the incidence of vector-borne disease via several mechanisms which reduce the mosquito population size and vector competence (CDC 2022). Here we will not explore the disease dynamics directly but will focus on the establishment and maintenance of Wolbachia infection in a mosquito population via cytoplasmic incompatibility (CI). CI means that eggs produced by females that are not Wolbachia-infected (hereafter referred to as wildtype) and fertilized by males that are Wolbachia-infected are not viable; eggs produced by Wolbachia-infected females and fertilized by any male are viable, and the majority of offspring are Wolbachia-infected. Therefore, in populations where Wolbachia is present, infected females have a reproductive advantage over uninfected females which, combined with vertical transmission, can drive increasing Wolbachia prevalence with each generation (Dorigatti et al. 2018; Hughes and Britton 2013; Sinkins 2004). The invasion of Wolbachia into a susceptible vector population is, however, not guaranteed. There is a trade-off between the reproductive advantage gained by infected mosquitoes through CI and a fitness cost they experience due to infection. Consequently, when Wolbachia is introduced into a susceptible population, it will only go to fixation (i.e. $100\%$ prevalence) if the initial prevalence exceeds a certain level known as the invasion threshold (Dorigatti et al. 2018).

Many cage and field experiments on establishing Wolbachia-infected mosquitoes in wildtype populations have been carried out over the past decade (Ross 2021; Dos Santos et al. 2022; Hoffmann et al. 2014; Dufault et al. 2022). Mathematical modelling has been used to support and explore the insights generated by these empirical studies. The majority of models neglect spatial structure and assume well-mixed populations (Hughes and Britton 2013; Ndii et al. 2012, 2015). Some models describe the movement of mosquitoes through space using dispersal kernels in reaction-diffusion frameworks (Turelli and Barton 2017; Hancock and Godfray 2012); others are highly complex simulations that admit limited analytical tractability (Magori et al. 2009; Hancock et al. 2019; Pagendam et al. 2020).

The dynamics of Wolbachia invasion at the scale of a town or city may be reasonably approximated by deterministic models such as these and formulated with ordinary or partial differential equations. However, Aedes aegypti mosquitoes often live in and around the same dwellings as the people they bite and the invasion dynamics unfold stochastically within small household mosquito populations. Wolbachia releases may even be conducted at the household scale with the objective of generating localised protection against dengue (O’Neill et al. 2018). In this study, we develop and analyse a stochastic model to investigate the invasion dynamics of Wolbachia-infected mosquitoes at the household scale. We use Markov process theory and matrix population theory to examine how the deterministic invasion threshold, which dictates whether an initial Wolbachia release spreads to the entire population, is characterised in a stochastic setting. We elucidate the medium and long term transient dynamics of the stochastic system to derive key quantities useful in Wolbachia release design, including the probability that Wolbachia-infected mosquitoes successfully invade the household, the expected time until invasion and the probability of reversion to a wildtype population.

The notion that stochastic effects can be important in the invasion dynamics of Wolbachia infection has previously been studied using Markov process frameworks based on classical population genetic models (Rigaud and Rousset 1996; Jansen et al. 2008; Turelli and Barton 2022) that assume finite, constant size populations. The stochastic invasion dynamics of Wolbachia were studied using the Wright-Fisher model by the authors of Rigaud and Rousset (1996). They derived an approximate expression for the probability of Wolbachia fixation following the introduction of a single Wolbachia-infected individual to wildtype population of size *N*. The authors of Jansen et al. (2008) derived an improved approximation for this fixation probability in very small populations, and derived exact results for specific demographic structures using the Moran model. They also considered the invasion dynamics arising from the introduction of multiple infected individuals, simultaneously or sequentially. They found that the fixation probability from the introduction of a single infected individual is proportional to $1/\sqrt{N}$ and can be substantial for small wildtype populations. For larger populations the corresponding fixation probability is very small but a steady influx of infected individuals can still lead to successful invasion on medium ecological timescales. These results led the authors to conclude that fixation in large wildtype populations may result from initial fixation in smaller, partially isolated populations. The approach we introduce here is complementary to the methods based on population genetic models. It makes use of theory for continuous-time Markov processes to focus on the invasion dynamics in very small, household-scale, populations that can fluctuate in size, and die out altogether. We explore the comparison between the two approaches further in the Discussion.

The structure of the paper is as follows. In Sect. 2, we review a deterministic mean-field model of wildtype and Wolbachia-infected mosquito population dynamics (Hughes and Britton 2013) in a household context and discuss the bistability of the steady states where only wildtype or only Wolbachia-infected mosquitoes are present. In Sect. 3, we recast the model in a stochastic framework and introduce the methods required to analyse the dynamics of Wolbachia invasion and persistence. In Sect. 4, we use a tutorial model with very small mosquito populations to elucidate the theory introduced in the previous section. In Sect. 5, we extend the model to incorporate more realistic bounds on the mosquito population size and investigate the invasion threshold and bistability in a stochastic setting. Finally, in Sect. 6, we consider the reversion of a Wolbachia-infected population to wildtype due to partially effective vertical transmission.

## Deterministic mean-field model of wildtype and Wolbachia-infected mosquito dynamics

Here we review a deterministic mean-field model for the dynamics of a population composed of wildtype and Wolbachia-infected mosquitoes. The model was first introduced by Hughes and Britton (2013). We consider a slightly simplified version given by

1 $$\begin{aligned} \frac{dN_m}{dt}&=b Z_m F(N_m + N_w) - dN_m, \end{aligned}$$

2 $$\begin{aligned} \frac{dN_w}{dt}&=b Z_w F(N_m + N_w) - d' N_w. \end{aligned}$$

Here, $N_m$ is the adult female wildtype mosquito population density and $N_w$ is the adult female Wolbachia-infected mosquito density. As in the original model, male population densities are implicitly assumed to be equal to the female densities. The per capita birth and death rates of the wildtype mosquitoes are denoted *b* and *d* respectively and the per capita death rate for the Wolbachia-infected mosquitoes is $d'=\delta d$, **where** $\delta \ge 1$. The quantities $Z_m$ and $Z_w$ are given by

3 $$\begin{aligned} Z_m=\frac{N_m}{N_m+N_w}(N_m+(1-v)\phi N_w) + \frac{N_w}{N_m+N_w}((1-u)N_m+(1-v)\phi N_w), \end{aligned}$$

4 $$\begin{aligned} Z_w=v\phi N_w. \end{aligned}$$

The $Z_m$ term gives the effective number of adults that produce wildtype offspring. The first term in (3) represents matings between wildtype males and wildtype or Wolbachia-infected males. It accounts for the fitness cost of infection, $1-\phi$, acting on reproduction and the proportion *v* of the offspring of infected females infected due to vertical transmission. The second term in (3) represents matings between Wolbachia-infected males and wildtype or Wolbachia-infected females. It accounts for a proportion *u* of the offspring of infected males and wildtype females that are non-viable due to CI. Wolbachia-infected females usually produce viable eggs regardless of the infection status of the male (Dorigatti et al. 2018). So we have slightly revised the expression for $Z_m$ given in Hughes and Britton (2013) to omit the CI effect from matings between infected males and infected females; their offspring are always viable. The $Z_w$ term gives the effective number of adults that produce infected offspring. It describes Wolbachia-infected females mating with any male to produce infected offspring. Hence $bZ_m$ and $bZ_w$ describe the maximum rates at which wildtype and Wolbachia-infected offspring are produced, in the absence of intraspecific competition, by $N_m$ wildtypes and $N_w$ Wolbachia-infected female mosquitoes.

The function *F*(*N*) in Eqs. (1)–(2) describes intraspecific competition between mosquito larvae. We use the heuristic function (Dye 1984)

5 $$\begin{aligned} F(N)={\left\{ \begin{array}{ll}\exp (-hN^k) & N < C,\\ 0 & N \ge C, \end{array}\right. } \end{aligned}$$

where *h* and *k* describe characteristics of larval competition. We have modified the model in Hughes and Britton (2013) such that the offspring rate is 0 when the adult population is *C* or more. In effect this means that the household female mosquito population cannot grow above *C*. Bounding the population size in this way ensures our stochastic model is tractable. Also note that in the original model the argument of the density dependence function is $N = Z_m + Z_w$, the effective size of the reproductive adult population after accounting for CI and the fitness costs of infection. However, we follow the same approach as Qu et al. (2022) and use $N = N_m + N_w$, the actual size of the adult population. For some formulations of the density dependence function *F*(*N*) this simplification can keep the system algebraically tractable. It does risk overestimating the impact of density dependence because the reproductive population is smaller than the adult population. However, the function *F* is heuristic with parameter flexibility that can accommodate the change in argument and, in the qualitative context of our conceptual model, this is a reasonable simplification.

A simpler alternative to *F*(*N*) is *G*(*N*) given by Qu et al. (2022)

6 $$\begin{aligned} G(N) = {\left\{ \begin{array}{ll} 1-N/C & N < C,\\ 0 & N \ge C. \end{array}\right. } \end{aligned}$$

**Fig. 1:**
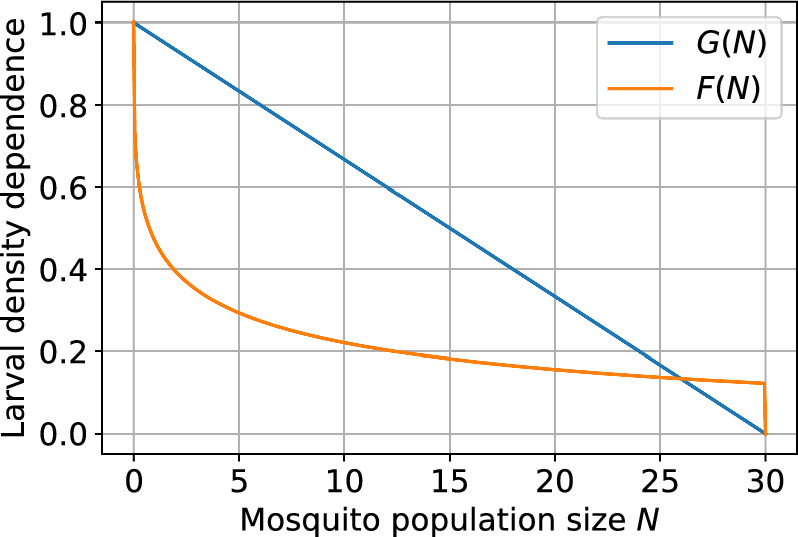
Larval intraspecific competition functions *F*(*N*) and *G*(*N*); see Eqs. (5) and (6). Parameter values are $C=30$, $k=0.3$ and $h=0.76$.

Since both functions are heuristic, in Sect. B in the Appendix, we reproduce our results using *G*(*N*) for comparison. Figure 1 shows the two larval intraspecific competition functions.

Model parameterisation is based on Hughes and Britton (2013). We have re-scaled the model such that $N_m$ and $N_w$ are in terms of mosquitoes per 100m^2^, the approximate area of a dwelling. We have fixed the reproductive carrying capacity, and effective upper bound on the female mosquito population size, at $C=30$. This bound lies well above observed mosquito household population sizes reported in the literature (Madewell et al. 2019). All parameter definitions and values are given in Table 1.

**Table 1:** Parameter definitions and values. Values except *C* and *b* are from Hughes and Britton (2013) and re-scaled so that mosquito population density is expressed per 100m^2^. All rates are per day. *C* is based on Madewell et al. (2019), *b* is calculated such that the wildtype female steady-state in the absence of any Wolbachia infection is $N_m^* = 10$.

Parameter	Definition	Value
*b*	Maximum per capita birth rate for wildtype mosquitoes.	$4.52d=0.54$ ($x^*=10$)
*d*	Per capita death rate of wildtype mosquitoes.	0.12
$b'=\phi b$	Maximum per capita birth rate for Wolbachia-infected mosquitoes.	$0.54\phi$
$\phi$	$1-\phi$ is the fitness cost of infection, which acts on reproduction. See $b^\prime$ above.	0–1
$d'=\delta d$	Per capita death rate of Wolbachia-infected mosquitoes.	0.12
$\delta$	Mortality cost of infection. See $d^\prime$ above.	1
*u*	Cytoplasmic incompatibility. Probability that offspring produced by a Wolbachia-infected male mating with a wildtype female are not viable.	1
*v*	Vertical transmission probability.	$0.9-1$
*C*	Reproductive carrying capacity.	30
*k*	Larval competition parameter.	0.3
*h*	Larval competition parameter.	$0.19\times 100^k=0.76$

### Stability analysis

We now summarise the steady state behaviour of the deterministic model (1)–(2). There are four steady state solutions: all mosquitoes absent $E_0=(N^*_m,N^*_w)=(0,0)$; only wildtype mosquitoes present $E_1=(F^{-1}(d/b),0)$; only Wolbachia-infected mosquitoes present $E_2=(0,F^{-1}(d'/(bv\phi )))$ and a coexistence state where both wildtype and Wolbachia-infected mosquitoes are present, $E_3$, which can be found numerically. If $b>d$, the absence steady state $E_0$ is unstable and $E_1$ is stable. If in addition $\phi b > \delta d$ then $E_2$ is also stable and the coexistence steady state $E_3$ is a saddle point (Hughes and Britton 2013).

**Fig. 2:**
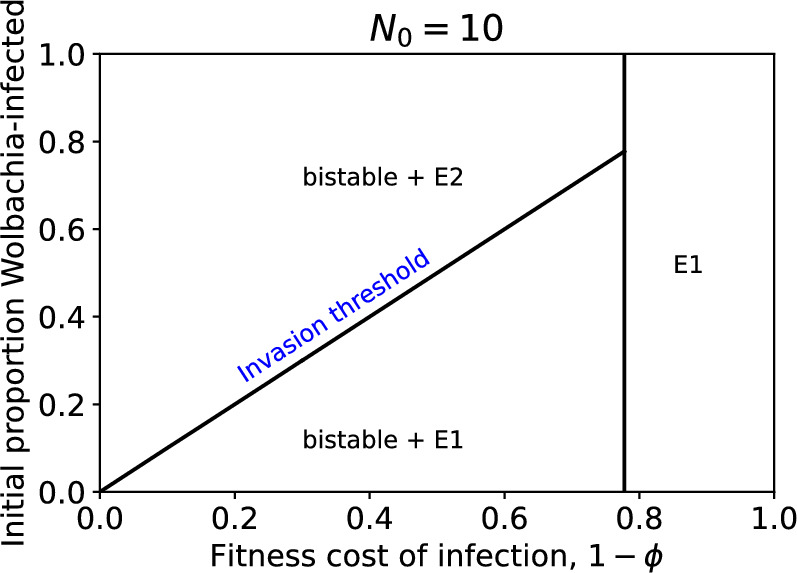
Stability and attraction of the steady states of system (1)–(2), with larval density function (5), depending on the fitness cost of Wolbachia infection $1-\phi$ and the initial proportion of the mosquito population that is Wolbachia-infected $N_w(0)/N_0$. Here $u,v=1$ and $N_0 = 10$. The diagram is divided along the horizontal axis into a region where the wildtype-only (*E*1) and Wolbachia-only (*E*2) steady states exist and are bistable, and a region where the unique non-trivial steady state is wildtype-only, and stable. The bistable region is further divided along the vertical axis into regions of initial conditions in the basins of attraction of *E*1 or *E*2. The invasion threshold in the bistable region is labelled in blue text. All of our stability analysis plots are produced using the open source software bSTAB Stender and Hoffmann (2022).

Linear stability analysis shows that system (1)-(2) is bistable in $E_1$ and $E_2$ when CI and vertical transmission are highly efficient i.e. *u* and *v* are both close to 1 Hughes and Britton (2013). This means that the outcome of introducing Wolbachia-infected mosquitoes into a household depends on the initial numbers of wildtype and infected mosquitoes in the household. Figure 2 shows how the stability of the wildtype-only and Wolbachia-only steady states, and their basins of attraction in terms of the initial proportion of the mosquito population that is Wolbachia-infected, depend on the fitness cost of infection. The invasion threshold is defined by the minimum proportion of the mosquito population that must be infected to be in the basin of attraction of the Wolbachia-only state. For a household with a total of $N_0=10$ (female) mosquitoes, the invasion threshold in terms of $N_w(0)/N_0$ is shown as a function of the reproductive fitness cost of Wolbachia infection $1-\phi \in [0,1]$. For our parameter values, the steady state (female) wildtype-only population is 10, so this threshold corresponds to the proportion of the wildtype mosquito population that would need to be replaced with Wolbachia-infected mosquitoes for eventual replacement of the entire wildtype population. When $1-\phi$ is above $1-d/b(=0.78)$, the fitness cost of infection is so high that the Wolbachia-only steady state is not biologically feasible. However, as $1-\phi$ decreases (and the fitness cost of infection reduces) the Wolbachia-only state becomes viable, and the invasion threshold decreases. The bistability is a key feature of the system. It shows that successfully establishing Wolbachia in a mosquito population requires the release of a sufficient number of infected mosquitoes, and that number depends on the fitness cost of infection associated with the Wolbachia strain.

**Fig. 3:**
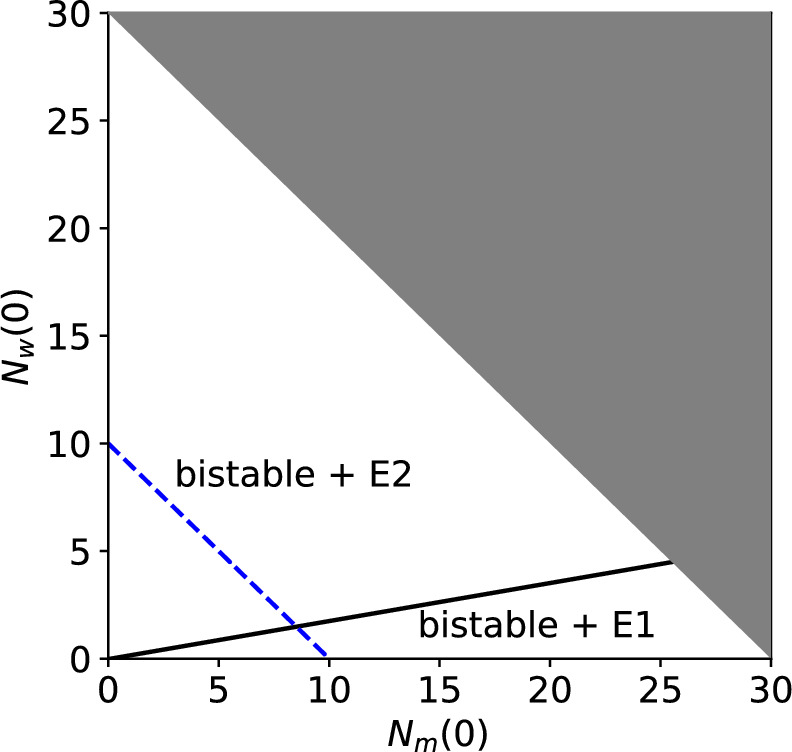
Stability and attraction of steady states of system (1)–(2) depending on the initial numbers of female wildtype $N_m(0)$ and Wolbachia-infected $N_w(0)$ mosquitoes. Here $u,v=1$ and $1-\phi =0.15$, with larval density function (5). Indicated on the diagram are the regions of initial conditions where solutions are in the basin of attraction of the wildtype-only (bistable + *E*1) or Wolbachia-only (bistable + *E*2) steady state. The grey triangle covers populations outside our upper bound on the total female mosquito population size. The dashed blue line indicates where $N_m(0)+N_w(0)=10$, corresponding to $1-\phi = 0.15$ in Fig. 2.

In Fig. 2 we assume the total female population size is fixed at $N_0 = 10$. We now relax this assumption. Figure 3 shows the basins of attraction in the bistable region when the initial numbers of (female) wildtype $N_m(0)$ and Wolbachia-infected $N_w(0)$ mosquitoes can take any values consistent with a total population size less than the reproductive carrying capacity ($C = 30$). In this figure, $1-\phi =0.15$ is fixed and the line $N_m(0)+N_w(0)=10$ corresponds to $\phi = 0.85$ in Fig. 2. We see that, regardless of the total population size, the Wolbachia-only steady state is reached if the proportion of Wolbachia-infected mosquitoes exceeds approximately 0.15.

## The stochastic framework

We now present a stochastic formulation of the model. The underlying structure is a continuous-time Markov chain (CTMC) $\mathcal {X}:=\{X(t),t\ge 0\}$ (Norris 1997; Anderson 2012) which describes the demographic evolution of the wildtype and Wolbachia-infected mosquito populations within a household. The household state is *X*. The state space is $\{(0,0)\} \cup \mathcal {S}$. Here $\mathcal {S}=\{(m,w) : 0< m+w\le C\}$ is a finite set of transient states, where *m* denotes the number of wildtype female mosquitoes and *w* denotes the number of Wolbachia-infected female mosquitoes in the household. As in the mean-field model in Sect. 2, we implicitly assume there is always an equal number of male mosquitoes. In order to keep the model computationally tractable, we impose an upper bound of *C* on the number of mosquitoes that can inhabit a household at a given time. State (0, 0) corresponds to the absence of both mosquito populations, and is absorbing since we assume that no external mosquito populations can be introduced to the household population. In alignment with the mean-field model, wildtype mosquitoes die at per capita rate *d*, Wolbachia-infected mosquitoes die at rate $\delta d$, wildtype mosquitoes are born at total rate $bZ_mF(m+w)$ and Wolbachiainfected mosquitoes are born at total rate $bZ_wF(m+w)$, where $Z_m$ and $Z_w$ are as in Eqs. (3)–(4). We interpret one unit of time as one day. The density dependent competition function *F* is configured so that $F(m+w) = 0$ if $m+w \ge C$, effectively bounding the population size at *C*. Table 2 summarises the transition rates for the model.

**Table 2:** Transition events and rates from household state *k* to state *l* in the CMTC model.

State *k*	State *l*	Transition	Rate
(*m*, *w*)	$(m+1,w)$	wildtype birth	$bZ_mF(m+w)$
(*m*, *w*)	$(m,w+1)$	Wolbachia-infected birth	$bZ_wF(m+w)$
(*m*, *w*)	$(m-1,w)$	wildtype death	*dm*
(*m*, *w*)	$(m,w-1)$	Wolbachia-infected death	$d\delta w$

In the interest of parsimony we do not allow movement between households. So our results can be interpreted in terms of a single isolated household, or a community of independent households. We assume that the offspring of Wolbachia-infected mosquitoes are always Wolbachia-infected (perfect vertical transmission, $v = 1$).

The CTMC describes how the state probabilities of the system evolve over time. It has transition (or generator) matrix $\boldsymbol{Q}_{0,0}$ with block matrix form Doorn and Pollett (2013)

7 $$\begin{aligned} \boldsymbol{Q}_{0,0}=\begin{pmatrix} 0 & \boldsymbol{0} \\ \boldsymbol{a}^T & \boldsymbol{Q} \end{pmatrix}, \end{aligned}$$

where $\boldsymbol{Q}=(q_{ij})$ is the transition matrix of the (sub) Markov chain which excludes the absorbing state (0, 0). We represent the states of $\mathcal {S}$ as single letters (rather than order pairs) to keep the notation concise. So entry $q_{ij}$ is the rate of transition from state *i* to state *j*, for $i \ne j$, and $q_{i,i}=-q_i=-\sum _{ i\ne j, j\in \mathcal {S} \cup \{(0,0)\}}q_{i,j}$. Note that in $\boldsymbol{Q}$, the probability dynamics of any state that communicates with the absorbing state, for example $i=(n,0)$ or (0, *n*) for $n \le C$, still includes the rate of transition to (0, 0). So $\boldsymbol{Q}$ does not conserve probability. The vector $\boldsymbol{a}=(a_i,\;i\in \mathcal {S})$ is composed of the rates of transition to the absorbing state. The $\boldsymbol{0}$ denotes a row vector of zeros of size $\vert \mathcal {S} \vert$.

Let $\boldsymbol{P}_{0,0}(t)$ be the row vector of the probabilities that a household is in each possible state at time *t*, with the first element corresponding to the absorbing state (0, 0). Then

8 $$\begin{aligned} \boldsymbol{P}_{0,0}(t)=\boldsymbol{P}_{0,0}(0)\exp {(\boldsymbol{Q}_{0,0}t)}. \end{aligned}$$

If $\boldsymbol{Q}_{0,0}$ is diagonalisable,

9 $$\begin{aligned} \boldsymbol{P}_{0,0}(t)=\sum _{i=1}^s c_ie^{-\lambda _it}\boldsymbol{l}_i \end{aligned}$$

where *s* is the cardinality of the state space, $-\lambda _i$ is the $i^{th}$ eigenvalue of $\boldsymbol{Q}_{0,0}$, $\boldsymbol{l}_i$ is its corresponding left eigenvector and the $c_i$ are constants (Keeling and Ross 2008). $\boldsymbol{Q}_{0,0}$ has all the eigenvalues of $\boldsymbol{Q}$ plus an additional eigenvalue 0 corresponding to the absorbing state. We can obtain a similar expression for $\boldsymbol{P}(t)$, the state probability vector of the transient subsystem, in terms of the eigenpairs of the transient state matrix $\boldsymbol{Q}$. Since the probability mass gradually moves from the transient states to the absorbing state (0, 0), which is not included in $\boldsymbol{P}(t)$, the sum of $\boldsymbol{P}(t)$ over all states will not generally equal 1 Doorn and Pollett (2013). However, as discussed in Mubayi et al. (2019) normalising $\boldsymbol{P}(t)$ so that it sums to 1 gives the state probability distribution conditional on non-extinction. Note that the matrix $\boldsymbol{Q}_{0,0}$ may not be diagonalisable, and we will not assume this in the proofs in Appendix I, but the expression in (9) is useful elsewhere.

### The quasi-stationary distribution

All the eigenvalues of $\boldsymbol{Q}$ have negative real part, indicating that the system eventually reaches the absorbing state (0, 0) and becomes trapped there. However, after the initial transient phase, but before extinction, the transient state probabilities of the system remain in a fixed ratio while probability accumulates in the absorbing state. In this phase the transient state probabilities conditioned on non-extinction are approximately stationary and their ratio is referred to as the quasi-stationary distribution (QSD) (Mubayi et al. 2019).

If $\boldsymbol{Q}$ is irreducible, it has a unique, simple and negative minimal magnitude eigenvalue $-\alpha$ (Doorn and Pollett 2013). Here minimal magnitude refers to the real part of the eigenvalue if it is complex. The QSD is given by the left eigenvector $\boldsymbol{l}$ associated with the eigenvalue $-\alpha$, normalised so that the elements sum to 1. The decay rate of the probability mass not already in the absorbing state is $\alpha$. If $\boldsymbol{Q}$ is reducible and the algebraic multiplicity of the minimal magnitude eigenvalue $-\alpha$ is 1 then the QSD is, as before, the left eigenvector of $\boldsymbol{Q}$ corresponding to $-\alpha$ normalised to sum to 1. If the algebraic multiplicity of $-\alpha$ is greater than 1, then the QSD is given by the eigenvalue-eigenvector pair of $\boldsymbol{Q}$ corresponding to the minimal communicating class of the state space $\mathcal {S}$ (Seneta 2006; Doorn and Pollett 2013). See Appendix A.1 for a more detailed account of this theory that may elucidate the constructions in Sect. 3.2.

### Communicating class structure of our model

A communicating class is a subset of the state space in which every pair of states *i* and *j* (in the concise notation) communicate, so *i* can be reached from *j* and vice-versa. The transient state space of our model where states are written out in full as ordered pairs are $\mathcal {S}=\{(m,w) : 0< m+w\le C\}$ consists of 3 communicating classes: $\mathcal {S}_{1}=\{(m,0):0 < m\le C\}$ is the class of wildtype-only states, $\mathcal {S}_{2}=\{(0,w):0< w \le C\}$ is the class of Wolbachia-only states and $\mathcal {S}_{3}=\{(m,w):m,w > 0 \;\; {\text {and}} \;\; m+w\le C\}$ is the class of mixed states. The mixed state class is labelled as $\mathcal {S}_{3}$ since the two other communicating classes are accessible from $\mathcal {S}_{3}$ but $\mathcal {S}_{3}$ is not accessible from $\mathcal {S}_{1}$ or $\mathcal {S}_{2}$.

The transient transition matrix $\boldsymbol{Q}$ can then be written in the form

10 $$\begin{aligned} \boldsymbol{Q}=\begin{pmatrix} \boldsymbol{Q}_1 & \quad \boldsymbol{0} & \quad \boldsymbol{0} \\ \boldsymbol{Q}_{21} & \quad \boldsymbol{Q}_2 & \quad \boldsymbol{0} \\ \boldsymbol{Q}_{31} & \quad \boldsymbol{Q}_{32} & \quad \boldsymbol{Q}_3 \end{pmatrix}, \end{aligned}$$

where $\boldsymbol{Q}_k$ denotes the block matrix composed of the transition rates between the states in $\mathcal {S}_{k}$ and $\boldsymbol{Q}_{ij}$ denotes the block matrix composed of the transition rates from the states in $\mathcal {S}_{i}$ to states in $\mathcal {S}_j$. Under the current framework, vertical transmission is perfect ($v = 1$) so there is no way to return from the Wolbachia-only state to a wildtype-only state (via some mixed state) and $\boldsymbol{Q}_{21}$ is a matrix of zeros. Note that the diagonal elements of $\boldsymbol{Q}_k$ are not equal to the negative sum of the rest of the corresponding row of $\boldsymbol{Q}_k$ because they include transitions to the absorbing state (0, 0). So, $\boldsymbol{Q}_k[i,i]= -\sum _{j \in \mathcal {S}\cup \{(0,0)\}} q_{i,j}$, where *i* corresponds to a given state in $\mathcal {S}_{k}$ and $q_{i,j}$ denotes the transition rate from state *i* to state *j* (in the concise state notation.

The absorbing state in our model can only be reached through $\mathcal {S}_{1}$ (wildtype-only states) or $\mathcal {S}_{2}$ (Wolbachia-only states). The probability mass initially drains out of $\mathcal {S}_{3}$ (mixed states) relatively quickly, then out of $\mathcal {S}_{1}$ or $\mathcal {S}_{2}$ to the absorbing state at a much slower rate. Since $v=1$, $\mathcal {S}_{1}$ and $\mathcal {S}_{2}$ are not communicating, so once the system enters $\mathcal {S}_{1}$ or $\mathcal {S}_{2}$ it remains in that class until extinction. The time to extinction is governed by the minimal magnitude eigenvalue of the relevant class transition matrix $\boldsymbol{Q}_1$ or $\boldsymbol{Q}_2$. Prior to extinction, the state probabilities in the class reach a stable distribution given by the left eigenvector associated with that eigenvalue. For further mathematical details, see Sect. I.2 in the Appendix.

### The probability of Wolbachia invasion

In Section 2.1 we saw that, in the deterministic model, there is a bistability between the wildtype-only and Wolbachia-only steady states. If the proportion of mosquitoes that are Wolbachia-infected exceeds the invasion threshold then, in the deterministic framework, a Wolbachia-infected population permanently replaces the wildtype population. We define a release that results in a household mosquito population becoming entirely Wolbachia-infected as ‘successful’. In the stochastic framework, exceeding the invasion threshold does not guarantee a successful release, and no mosquito population persists indefinitely. For any initial mixed state, the stochastic process eventually enters the absorbing state (0, 0). Before that, it passes through either the Wolbachia-only class or the wildtype-only class. A successful release must pass through the Wolbachia-only class.

Here, we will briefly discuss how to utilise standard Markov chain theory (Norris 1997) to calculate the probability of successful Wolbachia invasion. As before, let the wildtype-only, Wolbachia-only and mixed type classes be $\mathcal {S}_{1}$, $\mathcal {S}_{2}$ and $\mathcal {S}_{3}$ respectively. We denote the first time at which the Wolbachia-only class is entered as $T=\inf \{t\ge 0 :X(t)\in \mathcal {S}_{2}\}$, so that the probability of entering into the Wolbachia-only class at state $(0,w')\in \mathcal {S}_{2}$ having started in state $(m,w)\in \mathcal {S}_3$ is Gómez-Corral and López García (2012)

11 $$\begin{aligned} a_{(m,w)}(0,w')=\mathbb {P}(T<\infty , X(T)=(0,w')|X(0)=(m,w)). \end{aligned}$$

Clearly, the probability of entering the Wolbachia-only class at $(0,w')$ is 1 if the current state is $(0,w')$ and 0 if it is contained in $\mathcal {S}_{1}$, $\mathcal {S}_{2}\setminus \{(0,w')\}$ or if it is (0, 0).

Let $\boldsymbol{a}(0,w')=\left( a_{(m,w)}(0,w'): (m,w) \in \mathcal {S}_{3} \right)$ be a column vector of the probabilities of entering into $\mathcal {S}_{2}$ at $(0,w')$ from each state in $\mathcal {S}_{3}$. Let $\boldsymbol{q}(0,w')$ be a column vector containing the transition rates from each state in $\mathcal {S}_{3}$ to $(0,w')$. Then $\boldsymbol{a}(0,w')$ can be found by solving the linear system of equations:

12 $$\begin{aligned} -\boldsymbol{Q}_3 \boldsymbol{a}(0,w')=\boldsymbol{q}(0,w'). \end{aligned}$$

Here $\boldsymbol{Q}_3$ denotes the sub-*Q* matrix corresponding to the mixed household states in $\mathcal {S}_{3}$. The system of Eqs. (12) follows directly from Theorem 3.3.1 in Norris (1997), where the subset to be ‘hit’ (reached) is simply $\{(0,w')\}$. The same method can be used analogously to find the probabilities of entering the wildtype-only class at a given state from any given state in $\mathcal {S}_{3}$ by altering $\boldsymbol{a}$ and $\boldsymbol{q}$ accordingly. The probability of a successful Wolbachia invasion starting from a mixed state (*m*, *w*) is hence the sum of all the individual probabilities of entering $\mathcal {S}_{2}$ from $(m,w)\in \mathcal {S}_{3}$ via each of the states in $\mathcal {S}_{2}$.

### Time until Wolbachia invasion

Standard Markov chain theory (Norris 1997) can be extended to find the expected time until Wolbachia invasion. This is the time at which the household first enters a state in the Wolbachia-only class.

Let $\tau ^*_{(k,l)}$ be the expected time until the Wolbachia-only class $\mathcal {S}_{2}$ is reached from state $(k,l)\in \mathcal {S}_{2}\cup \mathcal {S}_{3}$ (a Wolbachia-only or mixed state), conditional on the event that $\mathcal {S}_{2}$ is reached from state (*k*, *l*). Let $a^*_{(k,l)}$ be the probability of reaching $\mathcal {S}_{2}$ from state (*k*, *l*). We determine the expected time until successful Wolbachia invasion originating from a mixed state in $\mathcal {S}_{3}$ by solving the linear system (Gómez-Corral and López García 2012):

13 $$\begin{aligned} \sum _{(k,l)\in \mathcal {S}_{2} \cup \mathcal {S}_{3}} q_{(m,w),(k,l)}a^*_{(k,l)}\tau _{(k,l)}^*=-a^*_{(m,w)}\;\;\; (m,w)\in \mathcal {S}_{3}. \end{aligned}$$

Recall that $\tau ^*_{(k,l)}=0$ for any $(k,l) \in \mathcal {S}_{2}$. That is, the time until a Wolbachia-only state is reached is 0 if the current household state is Wolbachia-only. Details of the derivation of Eqs. (13) are given in the Appendix H. The expected time until the wildtype-only class $\mathcal {S}_{1}$ is reached from any mixed state can be calculated analogously by taking $(k,l)\in \mathcal {S}_{1} \cup \mathcal {S}_{3}$.

### Quantifying the transient dynamics

Here we introduce some tools from matrix population theory (Caswell 2000) to calculate key quantities in our CTMC model. These quantities give indications of the expected time until the system reaches the QSD and the uncertainty associated with different regions of the state space.

We can derive the damping ratio $\rho$ from Eq. (9) as described in Caswell (2000); see Appendix G. Since we do not include the absorbing state in $\boldsymbol{P}(t)$, in our system the damping ratio quantifies the rate of convergence to the QSD and is defined as

14 $$\begin{aligned} \rho =\exp ((-\lambda _1)-(-\lambda _2)). \end{aligned}$$

The difference between the two eigenvalues is called the spectral gap and is often used to quantify the convergence of a Markov chain (Keeling and Ross 2008; Dambrine and Moreau 1981). We can therefore use the damping ratio as an indicator of how close the system is to the QSD.

The state entropy is another useful characterisation of the transient dynamics. Entropy measures how uncertain we are of the next state to be reached given we know the current state. For continuous-time processes (Spencer and Susko 2005) we calculate the entropy using the jump matrix of the CTMC which is composed of the probabilities $s_{i,j}$ that a process in state *i* moves next to state *j*

15 $$\begin{aligned} s_{i,j}={\left\{ \begin{array}{ll} -q_{i,j}/q_i & {\text {if }} i \ne j {\text { and }} q_i\ne 0,\\ 0 & {\text {if }} q_i=0, \end{array}\right. } \end{aligned}$$

and

16 $$\begin{aligned} s_{i,i} = {\left\{ \begin{array}{ll} 0 & {\text {if }} q_i \ne 0, \\ 1 & {\text {if }} q_i = 0. \end{array}\right. } \end{aligned}$$

Recall from Sect. 3 that $q_i$ is equivalent to $-q_{i,i}$. The entropy of state *i* is defined as

17 $$\begin{aligned} H_i= -\sum _{j\in \mathcal {S} \cup \{(0,0)\}} s_{i,j}\log {(s_{i,j})}. \end{aligned}$$

The state entropy accounts for the proportion of transitions that have non-zero rates, and their relative magnitudes (Hill et al. 2004). We will use the entropy to identify household states for which the dynamics are most uncertain because they give rise to larger numbers of possible stochastic pathways.

## Tutorial model - household with up to 3 female mosquitoes

**Fig. 4:**
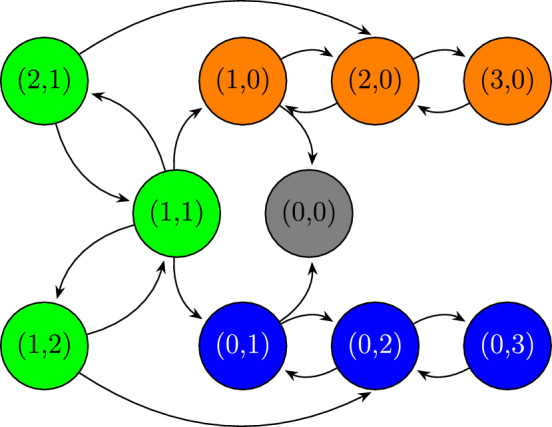
Schematic of the communicating classes in the 3 mosquito model. The states in the mixed class $\mathcal {S}_{3}$ are shaded green, the states in the wildtype-only class $\mathcal {S}_{1}$ are orange and the states in the Wolbachia-only class $\mathcal {S}_{2}$ are blue.

To aid understanding of the theory outlined above, in this section we work through a tutorial model. We set the upper bound on the number of (female) mosquitoes in the household at $C=3$. These mosquitoes can be wildtype or Wolbachia-infected. This configuration yields a small state space with just 9 transient states plus (0, 0), which facilitates visualisation of the communicating classes, the QSD and the associated dynamics. Figure 4 shows a state transition diagram which details the composition of the communicating classes outlined in Sect. 3.2. Although the maximum household size is much smaller than we would expect to observe in reality, the characteristic dynamics of the system are informative.

### Parameterisation

We parameterise the 3 mosquito model as in Table 1 except for setting $C=3$ and $b=0.3$ corresponding to a wildtype-only deterministic steady state of $x^*=2$. We also set the fitness cost of infection $1-\phi = 0.3$ and assume perfect transmission $v=1$. We chose these parameters to effectively illustrate how we use the mathematical techniques and interpret the transient dynamics of the system. In later sections, we will move towards a more realistic parameter regime supported by the literature (Hughes and Britton 2013). Larval competition parameters *h* and *k* remain the same as in Table 1. No reproduction occurs in households where the population has reached the upper bound.

### Results

We use the methodology described in Sect. 3 to determine the QSD and related metrics for the system with households of up to 3 mosquitoes. Table 3 shows the minimal magnitude eigenvalues of each of the communicating classes along with the quasi-stationary distribution derived from the eigenvector associated with overall minimal magnitude eigenvalue. This eigenvalue arises from the wildtype-only class. The second smallest eigenvalue in magnitude arises from the Wolbachia-only class. This indicates that the probability mass leaves $\mathcal {S}_{2}$ more quickly than it leaves $\mathcal {S}_{1}$. Consequently, for sufficiently large time, almost all of the probability mass outside of the absorbing state is in $\mathcal {S}_{1}$, and distributed according to the QSD. The QSD only holds non-zero probability mass in the states of the wildtype-only class. The minimal magnitude eigenvalue of class $\mathcal {S}_{3}$ is much larger than those of the other two classes. This indicates that the probability mass quickly moves out of the mixed class $\mathcal {S}_{3}$, then more slowly moves out of the wildtype-only and Wolbachia-only classes, $\mathcal {S}_{1}$ and $\mathcal {S}_{2}$, and into the absorbing state.

**Fig. 5:**
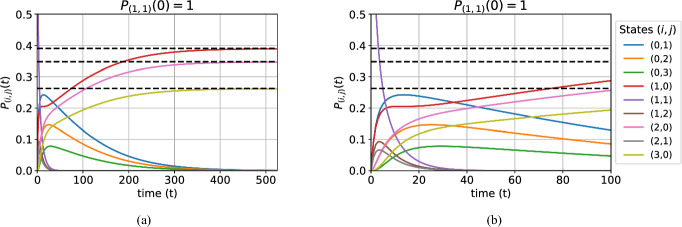
Probability distribution of all transient states over time, conditioned on non-extinction and initial state being (1, 1) with probability 1. Parameter values are as stated in Subsection 4.1. The legend details the transient state that each coloured probability distribution curve refers to in both figures. (a) Over 525 days and (b) enlargement of the first 100 days. The dashed lines indicate the QSD values for the wildtype-only states.

In Figs. 5a and 5b we show the dynamics of the transient state probability distribution over an interval of 525 days. We condition on non-extinction and an initial probability distribution $P_{(1,1)}(0)=1$ corresponding to one wildtype and one Wolbachia-infected (female) mosquito. The initial phase of the dynamics is most clear in Fig. 5b, which shows the first 100 days. The probability in the mixed states of class $\mathcal {S}_{3}$ quickly decays away in the first 40 days, accumulating in the wildtype-only or Wolbachia-only states of the $\mathcal {S}_{1}$ and $\mathcal {S}_{2}$ classes. In the next phase, shown in Fig. 5a, the probability mass decays out of the these classes and into the absorbing state. However, the decay is faster from $\mathcal {S}_{2}$ and the distributions are conditioned on non-extinction. Therefore, the conditioned probability mass in the Wolbachia-only states decays towards zero, and the probability mass in the wildtype-only states steadily increases towards to the QSD.

The QSD does not depend on the initial number of wildtype and Wolbachia-infected mosquitoes, so cannot be used to determine whether a release of Wolbachia-infected mosquitoes will successfully invade. The QSD is based on the probability mass dynamics of large ensembles of concurrent and independent realisations of the process, in our case corresponding to independent households. Any single realisation of the stochastic process will at some point either enter the wildtype-only class, and remain there until extinction, or enter the Wolbachia-only class and remain there until extinction. Within either of these classes, the probability mass decays to zero at a rate given by the minimal magnitude eigenvalue of the class and with stable state distribution given by the associated eigenvector (see Appendix Sects. I.1 and I.2 for a detailed justification).

**Table 3:** Key results for the model with households of up to 3 mosquitoes. Minimal magnitude eigenvalues of each class (see Sect. 3.2 for an explanation of the class structure), the probability of reaching the Wolbachia-only class (see Sect. 3.3), expected time to reach the Wolbachia-only class conditional on reaching the class (see Sect. 3.4), probability and expected time (conditional on reaching the class) to reach the wildtype-only class (see Sects. 3.3 and 3.4) and the QSD (see Sects. 3.1 and Appendix Sect. A.1). In the QSD vector, the first three terms correspond to the states (1, 0), (2, 0), (3, 0).

Result	Numerical values
Minimal magnitude eigenvalues for each communicating class	$-0.047$ $(\mathcal {S}_{1})$, $-0.052$ $(\mathcal {S}_{2})$, $-0.183$ $(\mathcal {S}_{3})$
Quasi-stationary distribution	[0.390, 0.348, 0.263, 0, 0, 0, 0, 0, 0]
Probability of reaching Wolbachia-only class from mixed state (*m*, *w*)	0.524 (1, 1), 0.682 (1, 2), 0.349 (2, 1)
Conditional expected time (days) to reach Wolbachia-only class from mixed state (*m*, *w*)	5.03 (1, 1), 5.35 (1, 2), 7.80 (2, 1)
Probability of reaching wildtype-only class from mixed state (*m*, *w*)	0.477 (1, 1), 0.318 (1, 2), 0.651 (2, 1)
Conditional expected time (days) to reach wildtype-only class from mixed state (*m*, *w*)	4.81 (1, 1), 7.59 (1, 2), 5.13 (2, 1)

Table 3 also gives the probabilities of reaching the Wolbachia-only class from each mixed state (*m*, *w*) with $m >0$ and $w >0$. We define this to be a successful invasion in the stochastic model context. The complements of these values are the probabilities of reaching the wildtype-only class. From (1, 1), the probability of reaching the wildtype-only, and the probability of reaching the Wolbachia-only class, are both close to 0.5. This is intuitive since the sequences of birth-death events required to reach either class are the same, and the probabilities of these sequences are similar when $\phi$ is close to 1. If the initial number of wildtype mosquitoes is larger than the number of Wolbachia-infected mosquitoes, the chance of reaching the wildtype class increases because the probability of wildtype births occurring is much larger than that of Wolbachia-infected births. The pattern is reversed if there are initially more Wolbachia-infected mosquitoes. The expected time required to reach the Wolbachia-only class from a mixed state is smallest from (1, 1) and largest from (2, 1). The expected time required to reach the wildtype-only class is also smallest from (1, 1) and largest from (1, 2).

The damping ratio calculated from the minimal eigenvalues of classes $\mathcal {S}_{1}$ and $\mathcal {S}_{2}$ allows us to estimate the convergence time to the QSD. The QSD is reached when the remaining probability mass in $\mathcal {S}_{2}$ is negligible compared to that in $\mathcal {S}_{1}$. The time until the total probability mass in $\mathcal {S}_{1}$ is 10 times that in $\mathcal {S}_{2}$ is $\log {(10)/\log {(\rho )}}$ (Caswell 2000). This value, along with the expected times for the probability mass in $\mathcal {S}_{1}$ to reach 100 and 1000 times that in $\mathcal {S}_{2}$ are shown in Table 5. Notice how these values are much larger, of the order several hundred days, than the expected time of around 6 days it takes to leave the mixed class $\mathcal {S}_{3}$ (shown in Table 3).

**Fig. 6:**
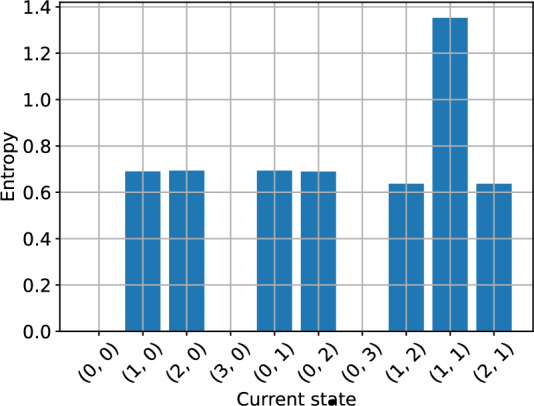
Individual entropies (uncertainty) of the next state to be reached given the current state. Evaluated in the model with households of up to 3 mosquitoes and parameter values as stated in Subsection 4.1.

The individual state entropies further elucidate the transient dynamics of the system. These quantities indicate how certain we are of the next state to be reached given the current state. In Fig. 6 values closer to 0 correspond to less variable dynamics whereas values closer to 1 indicate greater variability (Brice et al. 2020). There is no variability in the next state that is reached from (0, 3) and (3, 0) because the only possible event in each case is mortality. The entropy for state (0, 0) is 0 because it is absorbing. The highest variability arises from state (1, 1) because there are four possible birth/death events and the probabilities of birth/death events are similar in the wildtype and Wolbachia-infected populations. The entropies for states (1, 0), (2, 0), (0, 1) and (0, 2) are very similar because each of these states can reach two other states via a birth or a death event. However they do differ slightly due to the fitness cost $\phi$ acting on the Wolbachia-infected birth rate and density dependence effects via *F*. In comparison to these states, the entropies for states (1, 2) and (2, 1) are lower because the only possible events are wildtype or Wolbachia-infected mortality.

## Full model - households of up to 30 female mosquitoes

We now reparameterise the model so that a household can contain up to 30 female mosquitoes. This is a more realistic upper bound for a typical infestation. We choose parameters in such a way that in the deterministic model, the wildtype-only steady state is given by $x^*=10$. This value agrees with the mean-field model (1)–(2) from Sect. 2 scaled to household area. As before, we set the per capita birth rate *b* to achieve this steady state. Other parameters are in line with Hughes and Britton (2013) and given in Table 1. The fitness cost of Wolbachia infection $1-\phi$ is set to 0.15 unless otherwise stated. The vertical transmission probability is set to $v=1$, so that Wolbachia-infected mosquitoes can only produce infected offspring and there is no possibility of reversion from a Wolbachia-only state to a mixed state. The total transient state space $\mathcal {S}$ is decomposed as described in Sect. 3.2, where $\mathcal {S}_{1}=\{(m,0):0 < m\le 30\}$ is the wildtype-only class; $\mathcal {S}_{2}=\{(0,w):0< w \le 30\}$ is the Wolbachia-only class and $\mathcal {S}_{3}=\{(m,w):m,w > 0 \;\; {\text {and}} \;\; m+w\le 30\}$ is the mixed class.

A key aim of our analysis is to explore how stochasticity affects the transient and asymptotic dynamics previously identified in the deterministic system. In particular, we will examine how the deterministic bistability between the wildtype and Wolbachia-only steady states manifests in the stochastic model.

### Results

The minimal magnitude eigenvalues of each communicating class are shown in Table 4. As before, the overall minimal magnitude eigenvalue is associated with the wildtype-only class and the second minimal magnitude eigenvalue is associated with the Wolbachia-only class. The difference between these two eigenvalues is larger than the magnitude of the wildtype-only minimal magnitude eigenvalue, implying the QSD is valid. The QSD is shown in Fig. 16 in the Appendices. It only has non-zero probability mass in the wildtype-only states and is concentrated around the deterministic steady state $x^*=10$. The probabilities of household states with 28 or more female mosquitoes are close to 0, justifying our upper bound on household size. The damping ratio between classes $\mathcal {S}_{1}$ and $\mathcal {S}_{2}$ as the system approaches the QSD are shown in Table 5. In comparison with the model with households of up to 3 female mosquitoes (Sect. 4), the expected number of mosquitoes in the household is much larger (approximately 8 in the wildtype-only QSD, 5 in the Wolbachia-only QSD; see Appendix F) but the damping ratio indicates that the time to reach the QSD is only 2.4 times greater.

**Table 4:** Minimal magnitude eigenvalues of each communicating class in model with up to 30 female mosquitoes in a household, with reversion ($v=0.9$) and without reversion ($v=1$).

Model	$\mathcal {S}_{1}$	$\mathcal {S}_{2}$	$\mathcal {S}_{3}$
No reversion	$-0.002$	$-0.007$	$-0.079$
Reversion	$-0.002$	$-0.013$

**Table 5:** Damping ratio between classes $\mathcal {S}_{1}$ and $\mathcal {S}_{2}$, $\rho = \exp (\alpha _1 - \alpha _2)$ and the time until the probability mass in class $\mathcal {S}_{1}$ is 10, 100 and 1000 times the probability mass in $\mathcal {S}_{2}$ as the system approaches the QSD. Results are shown for the model with households of up to 3 female mosquitoes, up to 30 female mosquitoes with perfect vertical transmission (no reversion) and with imperfect vertical transmission (reversion).

Quantity	3 mosquitoes	30 mosquitoes no reversion	30 mosquitoes reversion
Damping ratio $\rho$	1.01	1.00	1.01
$\log (10)/\log (\rho )$	410	977	219
$\log (100)/\log (\rho )$	821	1954	438
$\log (1000)/\log (\rho )$	1231	2931	656

**Fig. 7:**
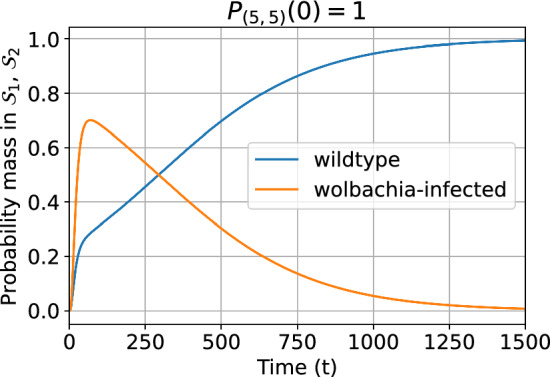
Dynamics of the probability distribution for the wildtype-only and Wolbachia-only classes conditioned on non-extinction. The class probability is the sum of the probabilities for all states in that class. For the model with households of up to 30 mosquitoes, perfect vertical transmission ($v=1$), $1-\phi =0.15$ and larval density function (5). Initially $P_{(5,5)}(0)=1$. For small *t* some of the probability mass is in the mixed class $\mathcal {S}_{3}$ but this quickly decays and has been omitted here.

Figure 7 shows the probability distribution over time for classes $\mathcal {S}_{1}$ and $\mathcal {S}_{2}$, conditioned on non-extinction. The initial household state is (5, 5) with probability 1. We chose (5, 5) as the initial condition since it has one of the largest individual state entropies (see Fig. 10) and so allows a broad array of possible stochastic trajectories. The mixed states do not hold significant probability mass for long and are disregarded (not shown in Fig. 7). The conditional probability mass in $\mathcal {S}_{2}$ initially grows faster than in $\mathcal {S}_{1}$ due to the reproductive advantage of Wolbachia infection conferred by CI. Each class then settles into a regime of exponential decay (to the absorbing state) with a stable state probability distribution well approximated by the QSD for the class in isolation. We provide some analytic insight into these dynamics in Appendix I.2. Conditioned on non-extinction as in the Fig. 7, the probability accumulates in the wildtype-only class as the fitness cost of infection drives extinction more quickly in the Wolbachia-only class.

**Fig. 8:**
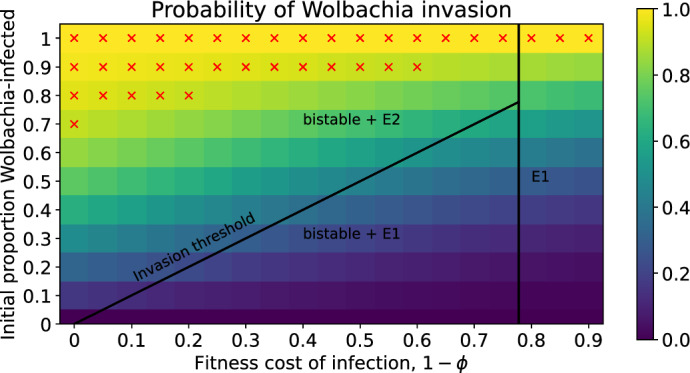
Probability of successful Wolbachia invasion of a household depending on the initial Wolbachia-infected proportion $N_w/N_0$ and fitness cost of infection $1-\phi$ with larval density function (5). The initial household size is fixed at $N_0=10$ female mosquitoes and the initial infected proportion is determined by $N_w(0)$ with $N_m(0) + N_w(0) = N_0$. Households can have up to 30 female mosquitoes and $v=1$. The solid lines and text show the stable steady states, basins of attraction and invasion threshold of the corresponding deterministic model. The red crosses indicate where the probability of successful Wolbachia invasion is greater than or equal to 0.9.

Figure 8 demonstrates how the deterministic bistability manifests in the stochastic model. Under the deterministic framework, we plotted the invasion threshold for Wolbachia-infected mosquitoes with household size $N_0=10$. This threshold separates the basins of attraction of the wildtype-only and Wolbachia-only steady states and is also shown in Fig. 8 for comparison. In the stochastic context, there is no such hard boundary. A Wolbachia invasion is ‘successful’ if the associated stochastic trajectory enters the Wolbachia-only class. Figure 8 shows the probabilities of successful Wolbachia invasions depending on the fitness cost of infection $1-\phi \in [0,0.9]$ and the initial proportion of Wolbachia-infected mosquitoes in the household. If the household contains a non-zero proportion of Wolbachia-infected mosquitoes there is a positive probability of successful Wobachia invasion. This probability decreases as the fitness cost of infection $1-\phi$ increases, and increases as the initial proportion of mosquitoes that are Wolbachia-infected increases. However, the probability of invasion is not constant along the deterministic invasion threshold. When the fitness cost of infection is low, the deterministic threshold suggests that only a small number of Wolbachia-infected mosquitoes are required for successful invasion. However, in the stochastic model, small Wolbachia-infected populations are prone to chance extinction and the probability of successful invasion from the initial population specified by the deterministic threshold is relatively low. The red crosses in Fig. 8 indicate where the probability of a successful Wolbachia invasion is greater than or equal to 0.9. Observe that when $1-\phi =0.15$, at least $80\%$ of the mosquitoes in the household need to be Wolbachia-infected in order for invasion to be successful with probability greater than or equal to 0.9. In Fig. 8 the total number of mosquitoes in the household is conserved while the proportion of Wolbachia-infected mosquitoes is varied. In Appendix C we show corresponding results when the household size is not conserved and the introduction of Wolbachia-infected mosquitoes may push the population size above the wildtype-only steady state.

**Fig. 9:**
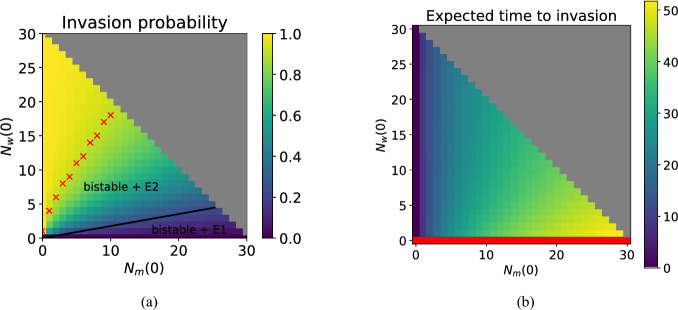
(a) Probability of entering the Wolbachia-infected class when the initial state is $(N_m(0), N_w(0))$. The solid black line is the corresponding deterministic threshold (Fig. 3). Red crosses denote, for different wildtype mosquito numbers, the minimum number of Wolbachia-infected mosquitoes required for successful invasion with probability 0.9 or higher. Note that, if there are more than 10 wildtype female mosquitoes present, the number of Wolbachia-infected mosquitoes required for a successful invasion with probability 0.9 or higher is outside of the state space. (b) Expected time in days to enter the Wolbachia-infected class, conditional on this event occurring, when the initial state is $(N_m(0), N_w(0))$. The red bar indicates initial conditions where only wildtype mosquitoes are present and so the Wolbachia-infected class cannot be reached. In both diagrams the maximum number of female mosquitoes in a household is 30, the initial state probability distribution is $P_{(N_m(0),N_w(0))}(0)=1$, parameters are as in Table 1, $1-\phi =0.15$, $v=1$, and the larval density function is (5). The top right quadrant shaded grey is not an admissible region of the state space.

Figure 9a shows how the probabilities of entering the Wolbachia-infected class depend on the initial state (*m*, *w*), which allows for resident populations above or below the steady state. As in Fig. 8, we overlay the corresponding deterministic boundary. Along the boundary line, we find that the probability of successful Wolbachia invasion stays fairly consistent; as long as there are at least 3 wildtype (female) mosquitoes in the household, the probability of invasion is close to 0.23. The red crosses indicate, for different wildtype mosquito numbers, the minimum number of Wolbachia-infected mosquitoes required for Wolbachia invasion to be successful with a probability greater than or equal to 0.9. Figure 9b shows how the expected time until Wolbachia invasion depends on the initial state. Larger wildtype populations increase the expected time required to enter the Wolbachia-infected class because it takes longer for them to die out. But, in all cases, the time for the invasion dynamics to resolve is of the order 50 days or less.

**Fig. 10:**
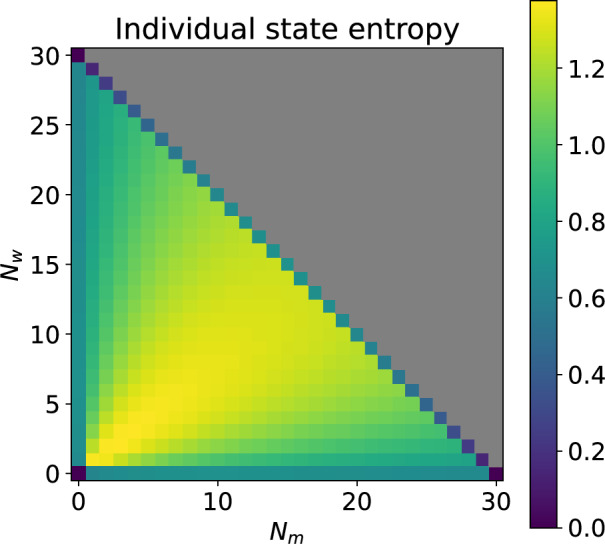
Entropy of state $(N_m,N_w)$. Higher entropy corresponds to greater uncertainty in the next state. The maximum number of female mosquitoes in household is 30, parameters are as in Table 1, $1-\phi =0.15$, $v=1$, larval density function is (5). The top right quadrant shaded grey is not an admissible region of the state space. There is some variation in the entropy along the first row and the first column of the diagram due to density dependence but it is too small to detect in the shading.

Figure 10 shows the state entropy for each state in $\mathcal {S}$. Larger entropy values correspond to a higher uncertainty in the next transient state the household will visit. Entropy is highest for the evenly mixed states in $\mathcal {S}_{3}$ because the number of possible events is maximised, and the event probabilities are evenly weighted. Entropy is lower for states in the wildtype-only or Wolbachia-only classes because the number of possible events is smaller. For the mixed states, the entropy is lowest at the maximum household size because the only possible events are wildtype or Wolbachia-infected mortality. The entropy is zero for the boundary states (30, 0) and (0, 30) since only a single mortality event is possible next. So the entropy may give an indication of how close the household is to the wildtype-only or Wolbachia-only class.

**Fig. 11:**
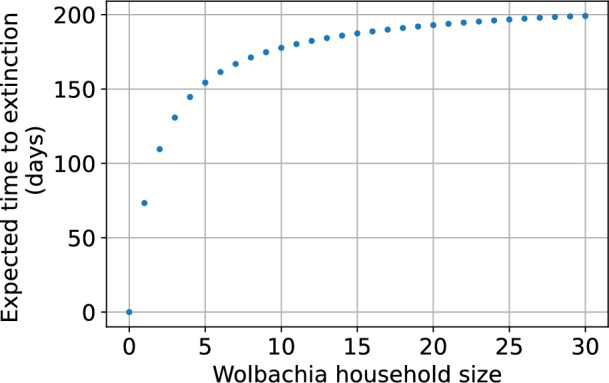
Expected time until extinction for a household population with no wildtype mosquitoes and up to 30 Wolbachia-infected female mosquitoes. Parameters are as in Table 1. The fitness cost of Wolbachia infection is $1-\phi =0.15$, $v=1$ and larval density function is (5).

In contrast to the deterministic model, in the stochastic framework a successful Wolbachia invasion does not establish the population permanently. The small population size and random walk of the population dynamics eventually lead to extinction and the household enters the absorbing state (0, 0). It is then vulnerable to repopulation by wildtype mosquitoes. Figure 11 shows the conditional expected times until extinction of Wolbachia-only household populations with up to 30 females calculated using a similar method to that described in Sect. 3.4; see Appendix E for more details. Expected extinction time increases with population size, but saturates. For population sizes at the deterministic steady state of 7 Wolbachia-infected female mosquitoes, the extinction time is 167 days, suggesting regular release may be needed to provide long term household protection.

In the next section we will consider imperfect vertical transmission, so the offspring of Wolbachia-infected females are wildtype with probability $1-v$, where $v < 1$. This means a household in a state in the Wolbachia-only class can move to a state in the mixed class and subsequently to the wildtype-only class. We will call this reversion. Note that reversion is a separate process to the complete extinction of Wolbachia-infected mosquitoes and subsequent reconolisation by a new population of wildtypes. However, the random walk of the Wolbachia-infected population towards extinction can work synergistically with imperfect vertical transmission to speed up reversion.

## Reversion to wildtype

### Model setup

If vertical transmission is imperfect ($v<1$) then some offspring of Wolbachia-infected mosquitoes are wildtype and states in $\mathcal {S}_{3}$ are accessible from $\mathcal {S}_{2}$, and vice-versa. So the state space $\mathcal {S}$ decomposes into two communicating classes. We relabel the communicating classes such that $\mathcal {S}_{1}=\{(m,0):0 < m\le 30\}$ contains the wildtype-only states and $\mathcal {S}_{2/3}=\{(m,w): w>0,\; 0<m+w\le 30\}$ contains the Wolbachia-only and mixed states. The full $\boldsymbol{Q}$ matrix in lower block triangular form is now

18 $$\begin{aligned} \boldsymbol{Q} = \begin{pmatrix} \boldsymbol{Q}_1 & \boldsymbol{0} \\ \boldsymbol{Q}_{2/3, 1} & \boldsymbol{Q}_{2/3} \\ \end{pmatrix}, \end{aligned}$$

where $\boldsymbol{Q}_1$ and $\boldsymbol{Q}_{2/3}$ are the sub-matrices representing the communicating classes $\mathcal {S}_{1}$ and $\mathcal {S}_{2/3}$ and $\boldsymbol{Q}_{2/3,1}$ contains the rates of transition from states in $\mathcal {S}_{2/3}$ to states in $\mathcal {S}_{1}$. The household is now able to escape the Wolbachia-only states into the mixed states, or the absorbing state, but remains unable to leave the wildtype-only class except to the absorbing state. We are interested in the probability that a household that has reached the Wolbachia-only class returns to the mixed class. We call this the reversion probability.

### Results

**Fig. 12:**
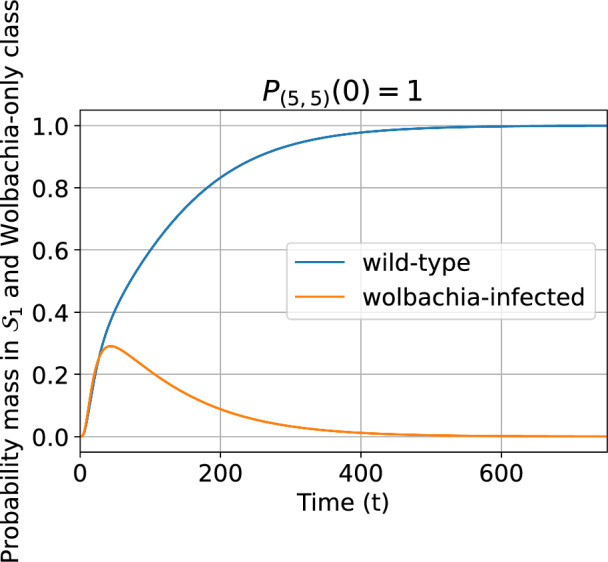
Dynamics of the probability distribution for the wildtype-only and Wolbachia-only classes conditioned on non-extinction when vertical transmission is imperfect. Note that the Wolbachia-only class is not a communicating class here. The class probability is the sum of the probabilities for all states in that class. For the model with households of up to 30 female mosquitoes, $v=0.9$, $1-\phi =0.15$, larval density function is (5). Initially $P_{(5,5)}(0)=1$.

We set the vertical transmission probability to $v=0.9$ but keep all other parameters as in Sect. 5. The minimal magnitude eigenvalues of the two communicating classes are given in Table 4. The overall minimal magnitude eigenvalue is unchanged, and associated with class $\mathcal {S}_{1}$, for any value of *v*. Since the dynamics of the chain within class $\mathcal {S}_{1}$ do not depend on *v*, the QSD is the same as for the model without reversion. However, the QSD is reached much more quickly than when $v=1$, see Fig. 12. This is because the leaky vertical transmission increases the effective reproduction rate for wildtype mosquitoes, and reduces it for Wolbachia-infected mosquitoes. Consequently, Wolbachia-only states move towards extinction more quickly.

The damping ratios for this model are given in Table 5. The state entropies are shown in Fig. 34 in Appendix D alongside results for the probability of Wolbachia invasion and the expected time to invasion. These quantities are very similar to the $v=1$ case.

#### Reversion probability

The reversion probability and expected time until reversion takes place can be used to estimate the duration of household protection and inform the release frequency of Wolbachia-infected mosquitoes. The probability of reversion can be found using the methodology described in Sect. 3.3. We now have two communicating classes and need to find a column vector $\boldsymbol{a}(m',0)=\left( a_{(m,w)}(m',0):(m,w)\in \mathcal {S}_{2/3}\right)$ containing the probabilities of entering the wildtype-only class at state $(m',0)$ from each state in $\mathcal {S}_{2/3}$ by solving the linear system

19 $$\begin{aligned} -\boldsymbol{Q}_{2/3}\boldsymbol{a}(m',0)=\boldsymbol{q}(m',0), \end{aligned}$$

where $\boldsymbol{Q}_{2/3}$ is the sub-Q matrix for $\mathcal {S}_{2/3}$ and $\boldsymbol{q}(m',0)$ is a column vector of the transition rates from each state in $\mathcal {S}_{2/3}$ to $(m',0)$. The probability of reaching $\mathcal {S}_{1}$ from a given state (*m*, *w*) in $\mathcal {S}_{2/3}$ is the sum of $a_{(m,w)}(m',0)$ over all $(m',0) \in \mathcal {S}_{1}$. We determine the total probability of reaching $\mathcal {S}_{1}$ from a given state distribution in $\mathcal {S}_{2/3}$ by using that distribution to construct a weighted sum of the probabilities of reaching $\mathcal {S}_{1}$ from each $(m,w) \in \mathcal {S}_{2/3}$.

**Fig. 13:**
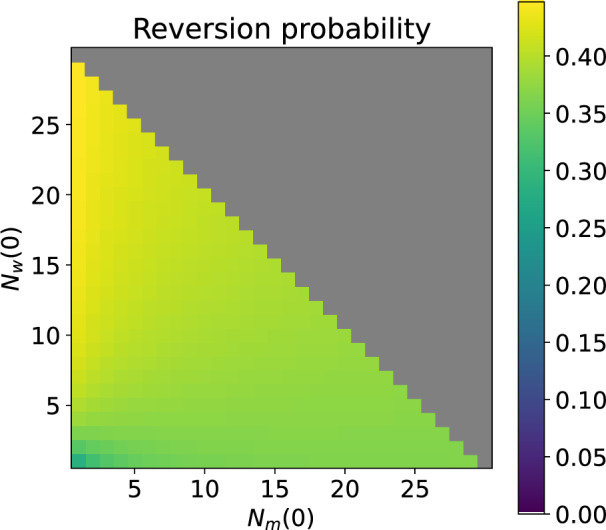
Probability of reversion from the Wolbachia-only states to the mixed states when a Wolbachia-only state is reached from an initial mixed state $(N_m(0), N_w(0))$. For the model with households of up to 30 mosquitoes, $v=0.9$, $1-\phi =0.15$, larval density function is (5).

To determine the state distribution of $\mathcal {S}_{2/3}$, consider that the wildtype-only class may be re-entered via any state in $\mathcal {S}_{2/3}$ and the Wolbachia-only states in $\mathcal {S}_{2/3}$ will have first passed through some mixed state (*m*, *w*). Therefore if we condition on starting at mixed state (*m*, *w*) with probability 1, it is possible to obtain the state distribution in the Wolbachia-only state space by considering the probabilities of entering each Wolbachia-only state (first out of all the Wolbachia-only states) via the mixed state (*m*, *w*). These probabilities are found by solving Eqs. (19) with $(m',0)$ swapped for Wolbachia-only state $(0,w')$ and only taking the rows and columns of $\boldsymbol{Q}_2$ corresponding to the mixed states. This allows us to perform an informal sensitivity analysis on the probability of reversion occurring within the household, conditioning on starting in the mixed state (*m*, *w*) via which the Wolbachia-only state space is first entered. This is presented in Fig. 13.

Giving equal weight to each possible initial mixed state, the average reversion probability when $1-\phi =0.15$ was 0.39. In the specific case where the Wolbachia-only class is reached from an initial mixed state (5, 5) the reversion probability is 0.38. In general, the reversion probability is slightly lower when starting from mixed states with smaller populations of mosquitoes or higher proportions of wildtype mosquitoes. In these cases, there is a higher probability that the Wolbachia-only class is entered via a state with low numbers of Wolbachia-infected mosquitoes and thus there is a higher risk of extinction before reversion is possible.

#### Expected time until reversion

**Fig. 14:**
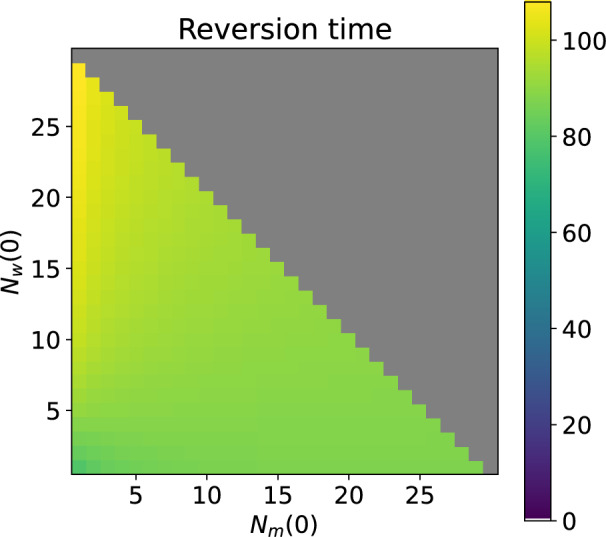
Expected time until reversion (in days) from the Wolbachia-only states to the mixed states (conditional on the event that reversion occurs) when a Wolbachia-only state is reached from an initial mixed state $(N_m(0), N_w(0))$. For the model with households of up to 30 mosquitoes, $v=0.9$ $1-\phi =0.15$, larval density function is (5).

We perform the same sensitivity analysis on the expected time until reversion (conditional on the event that reversion occurs) from a Wolbachia-only state to a wildtype-only state (Fig. 14), conditioning on each possible mixed state (*m*, *w*) initial condition. The average expected reversion time is 92 days and the reversion time conditional on the mixed state initial condition (5, 5) is 89 days. When the initial mixed state has a high proportion of Wolbachia-infected mosquitoes, the expected time until reversion after entering the Wolbachia-only class is slightly higher. This is because there is a higher probability that the Wolbachia-only class is entered via a state with high numbers of Wolbachia-infected mosquitoes. A large resident Wolbachia-infected population reduces the risk of extinction and, via density dependent competition and CI-induced inhibition of wildtype reproduction, the risk of re-invasion due to imperfect vertical transmission.

## Discussion

Ae. aegypti mosquitoes often live in and around the dwellings of the people that they bite, and may also be released at a household scale. So the invasion dynamics of Wolbachia-infection may be expected to unfold stochastically within small populations. In this study, we developed a stochastic model for the population dynamics of wildtype and Wolbachia-infected mosquitoes in a household setting. We used a continuous-time Markov chain framework aligned closely with a previously published mean-field model (Hughes and Britton 2013). Our overarching aim was to understand how deterministic bistability between wildtype-only and Wolbachia-only steady states, which determines whether a Wolbachia release leads to a successful Wolbachia invasion, is impacted by the inherent stochasticity of small populations.

Starting with a simplified tutorial model, we were able to utilise and adapt Markov and matrix population model theory to gain insights into the transient and asymptotic dynamics of the stochastic system. We calculated quantities such as the probability of Wolbachia invasion into a household after a release has been carried out, the expected time until invasion, and the quasi-stationary distribution of the system. This information provides estimates of the number of Wolbachia-infected mosquitoes that must be released in order to displace the wildtype population, and the frequency with which releases must be repeated in order to sustain the Wolbachia infection within the household. Such information will be useful to policy makers designing both community and household scale releases of Wolbachia-infected mosquitoes as an approach to control local mosquito populations. We focused on the impact of two key parameters: the fitness cost of Wolbachia infection on the mosquito’s birth rate, $1-\phi$, and the probability of vertical transmission of Wolbachia infection, *v*, which are known to vary between Wolbachia strains and have been shown in deterministic models to be important factors affecting the establishment of Wolbachia infection.

We studied the Wolbachia invasion probability under a range of initial population sizes of wildtype and Wolbachia-infected mosquitoes. We observed that the invasion probability is relatively consistent along the deterministic invasion boundary, but is quite small.

We compared frameworks that omit and allow reversion to a wildtype-only population after the Wolbachia-infected population has become established. This occurs via Wolbachia-infected females producing some wildtype offspring due to imperfect vertical transmission. Literature suggests the vertical transmission probability *v* is close to 1. We observed a difference in communicating class structure between the two frameworks. In reality, wildtype populations may be able to re-invade in either case if they migrate in from elsewhere, such as surrounding rural areas.

We found that introducing the minimum number of Wolbachia-infected mosquitoes required to entirely displace the wildtype population (ranging between 0 and 30 wildtypes) in the deterministic model only achieves that outcome approximately 23% of the time in the stochastic model ($v=1$). Successfully displacing the wildtype population at least 90% of the time requires the introduction of a much larger number of Wolbachia-infected mosquitoes. In the deterministic model we observe a hard boundary between the basins of attraction of the wildtype-only and Wolbachia-infected only steady states. But that boundary is blurred in the stochastic model because small populations of Wolbachia-infected mosquitoes often become extinct before proliferating sufficiently for cytoplasmic incompatibility to suppress the wildtype population. From a practical perspective, our results suggest that it may be necessary to consider releasing larger numbers of Wolbachia-infected mosquitoes into households than indicated by the invasion threshold of deterministic models. Multiple or ongoing releases may be required before successful invasion into, and replacement of, the wildtype population.

The stochastic invasion dynamics of Wolbachia have previously been examined using Wright–Fisher and Moran models (Rigaud and Rousset 1996; Jansen et al. 2008). Our modelling approach complements these earlier studies by taking full account of the stochastic dynamics of very small populations. Our Markov process framework elucidates the underlying population dynamic mechanisms, in particular the population structures of the invasion process expressed through the probability dynamics of the communicating classes. A key distinction of our method is that it does not impose the restriction of constant population size and extinction is therefore an inevitable outcome. This feature is particularly important in the context of very small populations, such as those typically found in households, where localized invasion, extinction and re-colonization govern the dynamics of both wildtype and Wolbachia-infected mosquitoes. Despite these differences, the principal results of our analysis summarised above are broadly aligned with those reported by the authors of Jansen et al. (2008).

**Fig. 15:**
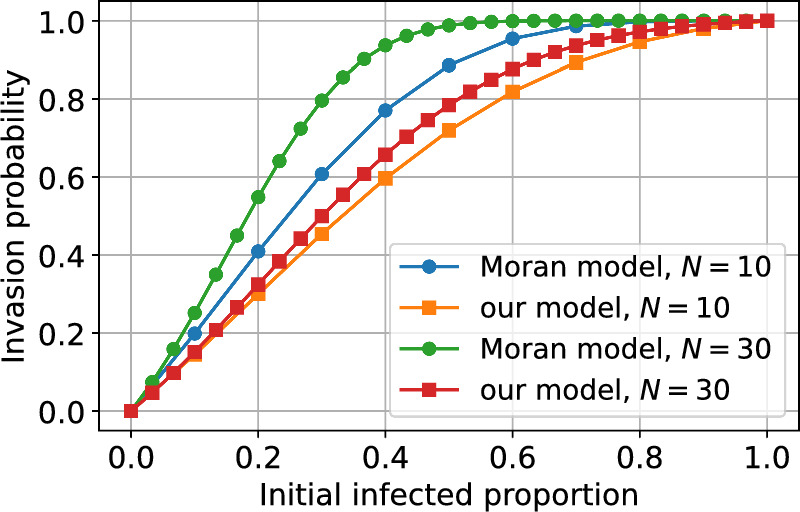
The probability of a successful Wolbachia invasion for initial population size $N=N_m(0)+N_w(0)$ dependent on the initial infected proportion $N_w(0)/N$. The legend details the model framework used and the initial total population size for each curve. The invasion (fixation) probability for the Moran model was calculated using the function $u(p_0)$ given in Jansen et al. (2008) with parameterisation and mosquito reproduction modified to align with our model ($\theta _u = bF(N_m+N_w)Z_m, \theta _i =bF(N_m+N_w)Z_w$) All parameter values can be found in Table 1, where we assume $v=1$ and use the larval density function (5).

**Fig. 16:**
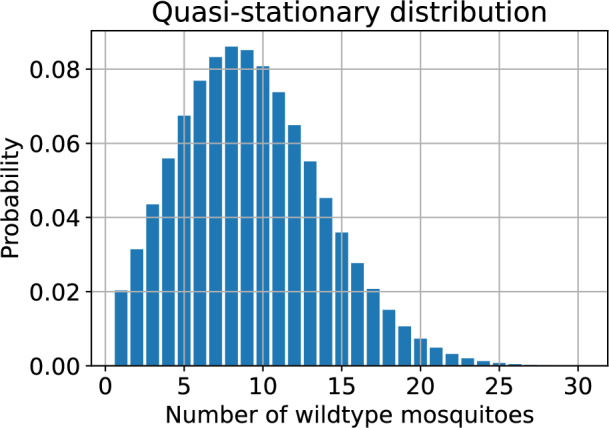
QSD for the model with households of up to 30 female mosquitoes, perfect vertical transmission ($v=1$), $1-\phi =0.15$ and larval density function (5). Any states not shown have probability equal to 0.

**Fig. 34:**
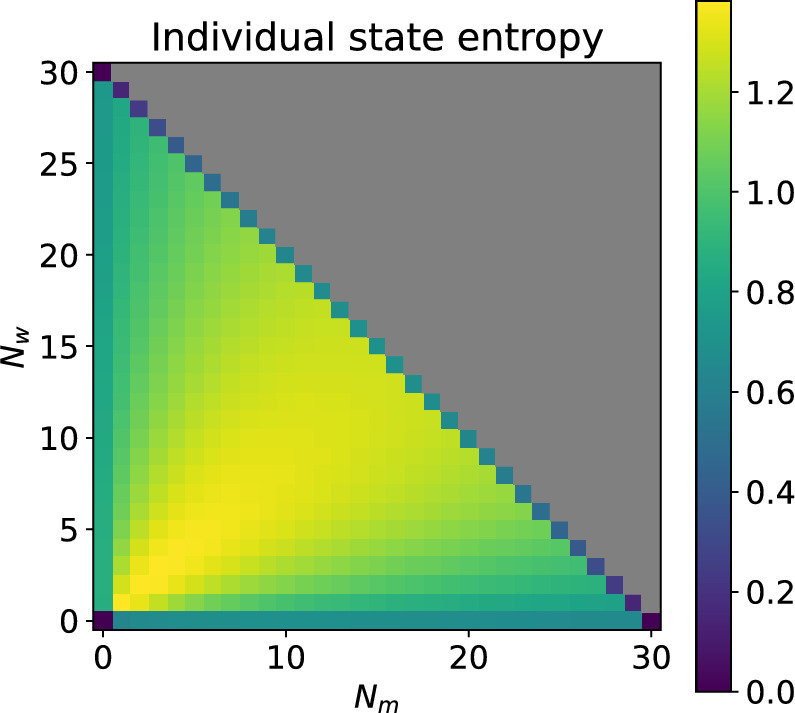
Entropy of state $(N_m,N_w)$. Higher entropy corresponds to greater uncertainty in the next state. The maximum number of female mosquitoes in household is 30, parameters are as in Table 1, $1-\phi =0.15$, $v=0.9$, larval density function is (5). The top right quadrant shaded grey is not an admissible region of the state space.

For a specific comparison, we show the fixation (i.e. successful invasion) probability as a function of the initial infection proportion for both approaches in Fig. 15. For the Moran model, we used the fixation probability $u(p_0)$ as given in Jansen et al. (2008) with the functions that give the number of offspring modified to account for our parameterisation and formulation of cytoplasmic incompatibility. The models produce a broadly similar relationship between the probability of successful invasion and the initial infected proportion. However, under the Moran model, the fixation/invasion probability increases and saturates more rapidly than in our model. This discrepancy suggests that in very small mosquito populations, stochastic variation in the total population size can substantially influence invasion probability. In particular, large wildtype populations may dilute the infected proportion and hinder invasion.

Our robust analysis of the stochastic dynamics of Wolbachia invasion in a single household lays the groundwork for stochastic invasion models based on communities of households connected by mosquito dispersal. We found another study (Magori et al. 2009) that considered stochastic effects on mosquito population dynamics under the finer scale of mosquito breeding habitats or households. The framework is a highly complex agent based model that admits limited analytical tractability and does not incorporate Wolbachia releases as a control strategy. The Wright-Fisher derived model (Rigaud and Rousset 1996; Jansen et al. 2008) has, however, been employed effectively at a coarser scale to analyse Wolbachia invasion data from North Queensland, Australia (Turelli and Barton 2022). The data record Wolbachia successfully invading a community then slowly but persistently dispersing into a nearby community separated by a major road. The second community was only successfully invaded after several years. The model analysis found that the data could be explained by either stochastic or deterministic dynamical processes. Were household scale data to become available it would be insightful to perform a similar analysis using a household structured model.

We acknowledge several limitations and assumptions in our model. We only explicitly modelled the female mosquito population, and assumed there are an equal number of males in the population. This approximation is in line with deterministic models (Hughes and Britton 2013; Qu et al. 2022) and is reasonable because we examine only the simple setting of a single household, with the objective of realising the mosquito invasion dynamics at a household level. However, male only releases are a common control strategy for mosquito population suppression, acting similarly to the sterile insect technique (Ross 2021). Male only releases have been studied by the authors of Pagendam et al. (2020) using a continuous time Markov process model. They investigate the impact of unintentional releases of small numbers of Wolbachia-infected females under a male-only suppression strategy release. They accounted for non-exponentially distributed mosquito lifetimes by incorporating a number of different classes of individuals. They concluded that it is beneficial to use regular male releases that adapt to the decreasing wildtype population size in order to minimize the risk of releasing females and reduce costs.

We imposed an upper bound on the female population size in the household of 30 mosquitoes. This assumption is necessary because otherwise the state space $\mathcal {S}$ would become infinite. Although we suspect many of our analytical quantities would still hold under an infinite state space $\mathcal {S}$, the numerical results from this study would become unattainable. Observed household mosquito populations reported in the literature suggest this upper bound is reasonable (Madewell et al. 2019). We only considered the invasion dynamics in a single household or, equivalently, a community composed of a large number of independent households. This assumption allows analytic tractability and ensures the state space is computationally manageable. However, movement of mosquitoes between households may be an important factor in the establishment and maintenance of Wolbachia infection at the household and community scales.

We have assumed that offspring produced by a Wolbachiainfected male mating with a wild type female are not viable, so $u=1$. This assumption is supported by empirical work involving thousands of Ae. aegypti eggs that reported near complete CI (Hoffmann et al. 2014) and is widely adopted in modelling studies (Jansen et al. 2008; Hughes and Britton 2013; Turelli and Barton 2017). It is possible that incomplete CI may arise for some Wolbachia strains, or between mosquitoes infected with distinct Wolbachia strains. If $u<1$ then matings between Wolbachia-infected males and wildtype females produce some viable offspring. The total wildtype reproduction rate will be higher, Wolbachia-infected reproduction will be unchanged. So we would expect the deterministic invasion threshold to be higher and, in the stochastic model, the probability of successful Wolbachia invasion to be lower. The details of this relationship may be an interesting avenue for further study, possibly building on recent work in Ma and Su (2024); Orozco-Gonzales et al. (2024).

In a follow up study (Barlow and Adams 2025) we have addressed some of these issues and further explored practical aspects of Wolbachia releases for local and community mosquito control. We extended the single household framework to a system of connected households. This required the incorporation of movement of mosquitoes between households. Male and female Ae. aegypti movement behaviour is contrasting. Males tend to move around more frequently in search of mates (Trewin et al. 2021), moving distances of around 200 to 400 metres over their lifetime (Juarez et al. 2020; Marcantonio et al. 2019; Trewin et al. 2020), whereas females typically move less than 100 metres in their lifetime. This distinction reinforces the need to consider differentiating between male and female mosquitoes. It is also possible that sex ratios among wildtype and infected mosquito types may become skewed, altering the reproductive rates and driving qualitative changes in the invasion dynamics. A community of connected households may also affect the bistability result reported for a single population if there is a larger set of possible steady state solutions. Incorporating movement between households into our CTMC model introduced nonlinearities related to community scale dispersal to household scale containers. This complexity stymied many of the analytic approaches discussed in this study, and necessitated the use of stochastic simulation methods (Gillespie 1977; Barlow et al. 2025).

We defined a larval density dependence function acting on the birth rate, which describes the effect of intraspecific competition for resources such as food and space. We investigated two common larval density dependence functions (Dye 1984; Qu et al. 2022). Both of these models are heuristic, although some data was used to estimate parameters in Eq. (5) under the original scaling (Hughes and Britton 2013; Dye 1984). The overall results of our analysis are qualitatively similar for both models. The main difference is that Eq. (5) produces a much higher birth rate than (6), leading to the Wolbachia-only steady state existing (in the mean-field model) for a larger region of the Wolbachia fitness parameter space. Were more data to become available, it would beneficial to revisit the larval density dependence function used here and in other studies (Dye 1984; Hughes and Britton 2013; Ndii et al. 2012; Qu et al. 2022).

In this study we have concentrated on the mosquito invasion dynamics alone. But the overarching motivation for establishing Wolbachia infection in domestic mosquitoes is to reduce the circulation of dengue and other vector-borne diseases. In many cases the host-vector transmission dynamics of those infectious diseases will also take place at a household scale and differences in human household sizes and commuter patterns could act to transform the mosquito invasion dynamics. There has been some progress in modelling vector-borne disease dynamics at the household scale (Black et al. 2022). An obvious next step would be to develop that framework to include Wolbachia infection.

In conclusion, Aedes aegypti mosquitoes favour urban residential habitats. The mosquito population dynamics, and efforts to control them, unfold stochastically at a household scale. Deterministic models have demonstrated that releasing Wolbachia-infected mosquitoes can displace the wildtype population, as long as a sufficient number of infected mosquitoes are introduced. Stochastic models support this inference, but suggest that the relationship between the initial introduction and the eventual outcome is more nuanced.

## Supplementary information

All code used to produce the results presented in this paper can be found at https://github.com/ahb48/Wolbachia_invasion_households.

## Figures and Tables

**Fig. 17:**
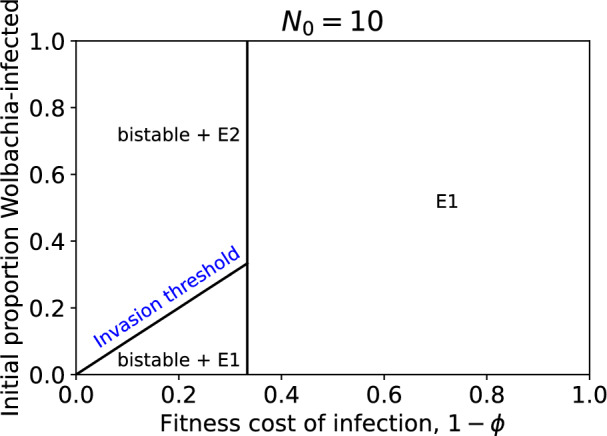
Stability and attraction of the steady states of system (1)–(2), with larval density function (6), depending on the fitness cost of Wolbachia infection $1-\phi$ and the initial proportion of the mosquito population that is Wolbachia-infected $N_w(0)/N_0$. Here $u,v=1$ and $N_0 = 10$. The diagram is divided along the horizontal axis into a region where the wildtype-only (*E*1) and Wolbachia-only (*E*2) steady states exist and are bistable, and a region where the unique non-trivial steady state is wildtype-only, and stable. The bistable region is further divided along the vertical axis into regions of initial conditions in the basins of attraction of *E*1 or *E*2. The invasion threshold under the bistable region is labelled in blue text. All of our stability analysis plots are produced using the open source software bSTAB (Stender and Hoffmann 2022).

**Fig. 18:**
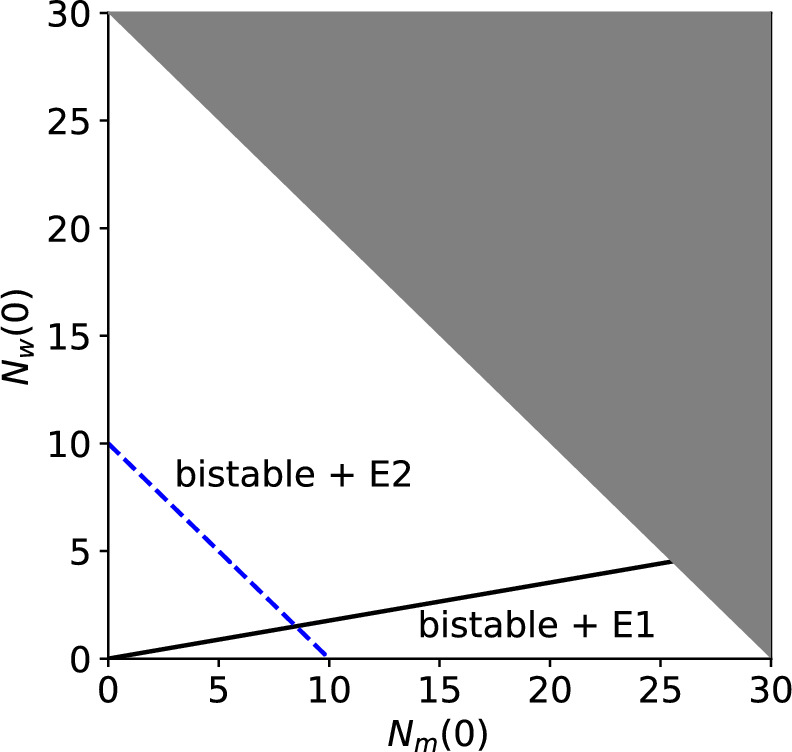
Stability and attraction of steady states of system (1)–(2) depending on the initial numbers of female wildtype $N_m(0)$ and Wolbachia-infected $N_w(0)$ mosquitoes. Here $u,v=1$ and $1-\phi =0.15$, with larval density function (6). Indicated on the diagram are the regions of initial conditions in the basins of attraction of the wildtype-only (*E*1) or Wolbachia-only (*E*2) steady state. The grey triangle covers populations outside our upper bound on the total female mosquito population size. The dashed blue line indicates where $N_m(0)+N_w(0)=10$, corresponding to $1-\phi = 0.15$ in Fig. 17.

**Fig. 19:**
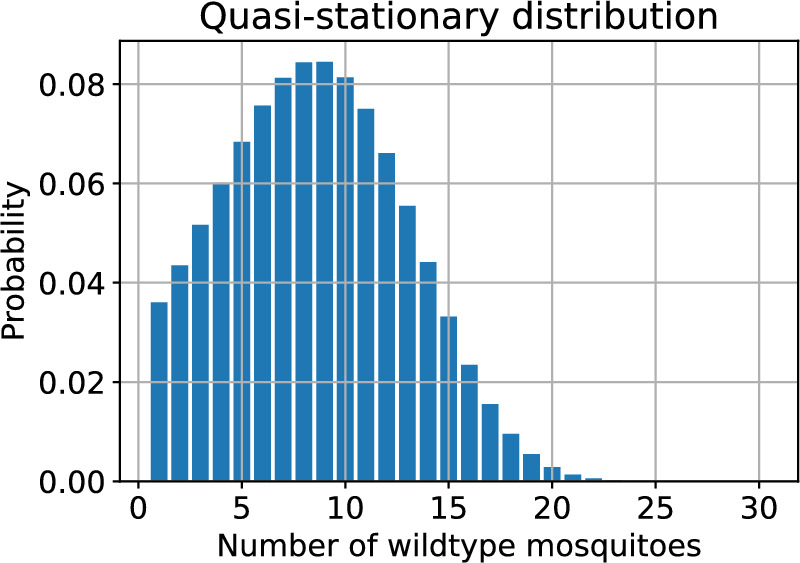
QSD for the model with households of up to 30 female mosquitoes, perfect vertical transmission ($v=1$), $1-\phi =0.15$ and larval density function (6). Any states not shown have probability equal to 0.

**Fig. 20:**
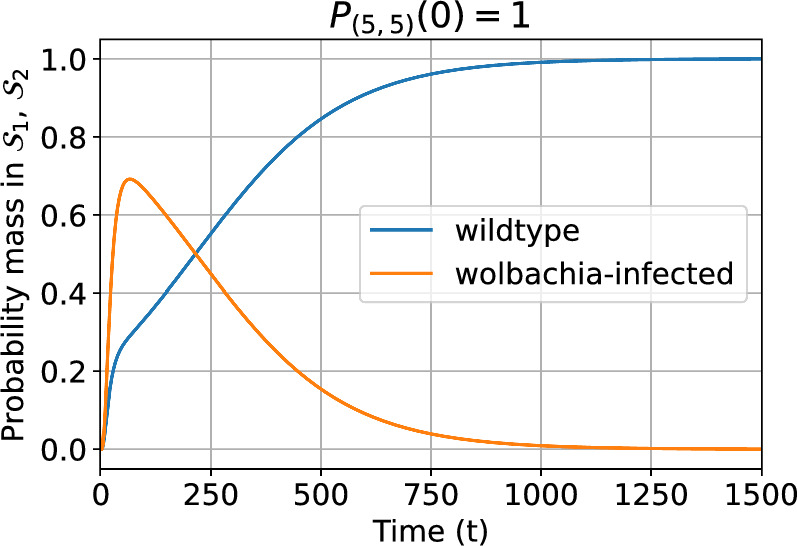
Dynamics of the probability distribution for the wildtype-only and Wolbachia-only classes conditioned on non-extinction. The class probability is the sum of the probabilities for all states in that class. For the model with households of up to 30 mosquitoes, perfect vertical transmission ($v=1$), $1-\phi =0.15$ and larval density function (6). Initially $P_{(5,5)}(0)=1$. For small *t* some of the probability mass is in the mixed class $\mathcal {S}_{3}$ but this quickly decays and has been omitted here.

**Fig. 21:**
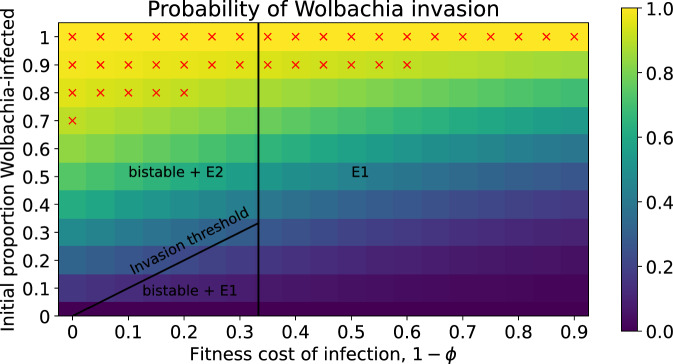
Probability of successful Wolbachia invasion of a household depending on the initial Wolbachia-infected proportion $N_w/N_0$ and fitness cost of infection $1-\phi$ with larval density function (6). The initial household size is fixed at $N_0=10$ female mosquitoes and the initial infected proportion is determined by $N_w(0)$ with $N_m(0) + N_w(0) = N_0$. Households can have up to 30 female mosquitoes and $v=1$. The solid lines and text show stable steady state, basins of attraction and invasion threshold of the corresponding deterministic model. The red crosses indicate where the probability of successful Wolbachia invasion is greater than or equal to 0.9.

**Fig. 22:**
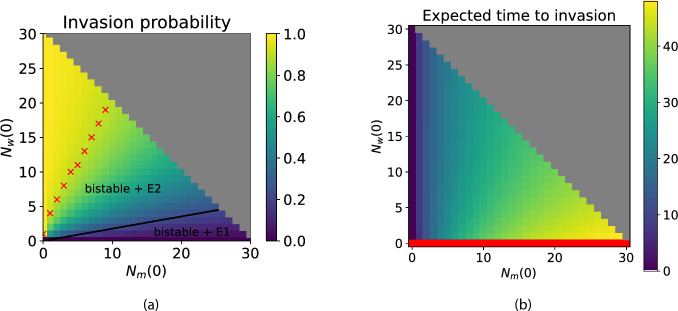
(a) Probability of entering the Wolbachia-infected class when the initial state is $(N_m(0), N_w(0))$. The solid black line is the corresponding deterministic threshold (Fig. 18). Red crosses denote, for different wildtype mosquito numbers, the minimum number of Wolbachia-infected mosquitoes required for successful invasion with probability 0.9 or higher. Note that, if there are more than 9 wildtype female mosquitoes present, the number of Wolbachia-infected mosquitoes required for a successful invasion with probability 0.9 or higher is outside of the state space. (b) Expected time in days to enter the Wolbachia-infected class, conditional on this event occurring, when the initial state is $(N_m(0), N_w(0))$. The red bar indicates initial conditions where only wildtype mosquitoes are present and so the Wolbachia-infected class cannot be reached. In both diagrams the maximum number of female mosquitoes in a household is 30, the initial state probability distribution is $P_{(N_m(0),N_w(0))}(0)=1$, parameters are as in Table 1, $1-\phi =0.15$, $v=1$, and the larval density function is (6). The top right quadrant shaded grey is not an admissible region of the state space.

**Fig. 23:**
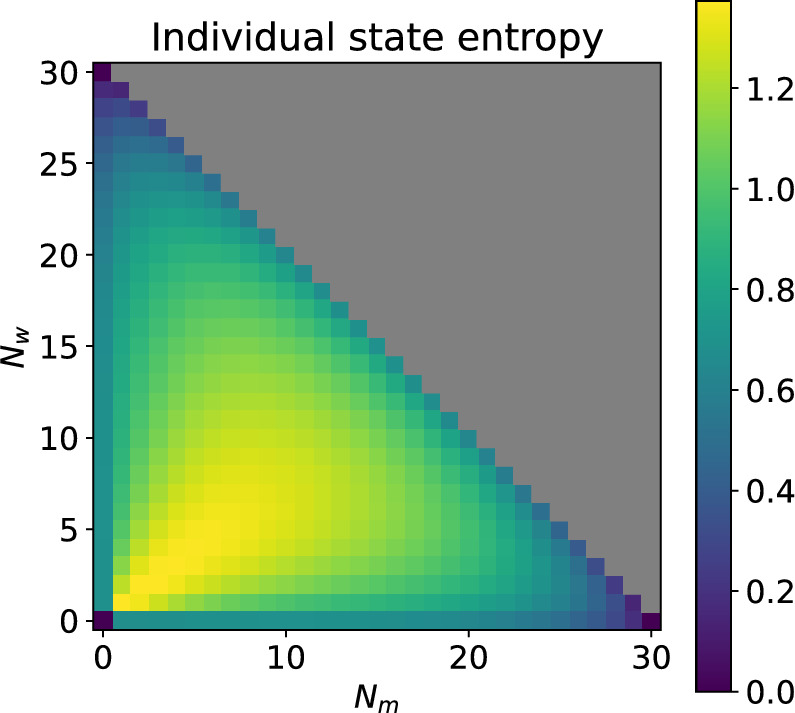
Entropy of state $(N_m,N_w)$. Higher entropy corresponds to greater uncertainty in the next state. The maximum number of female mosquitoes in household is 30, parameters are as in Table 1, $1-\phi =0.15$, $v=1$, larval density function is (6). The top right quadrant shaded grey is not an admissible region of the state space.

**Fig. 24:**
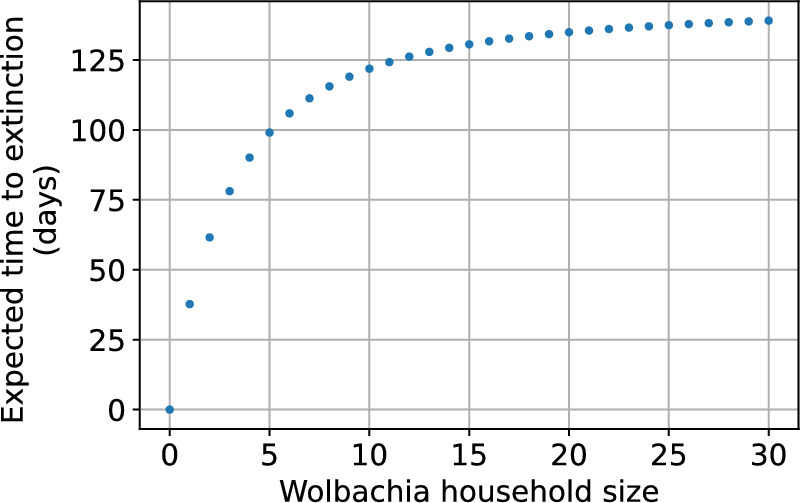
Expected time until extinction for a household population with no wildtype mosquitoes and up to 30 Wolbachia-infected female mosquitoes. Parameters are as in Table 1. The fitness cost of Wolbachia infection is $1-\phi =0.15$, $v=1$ and larval density function is (6).

**Fig. 25:**
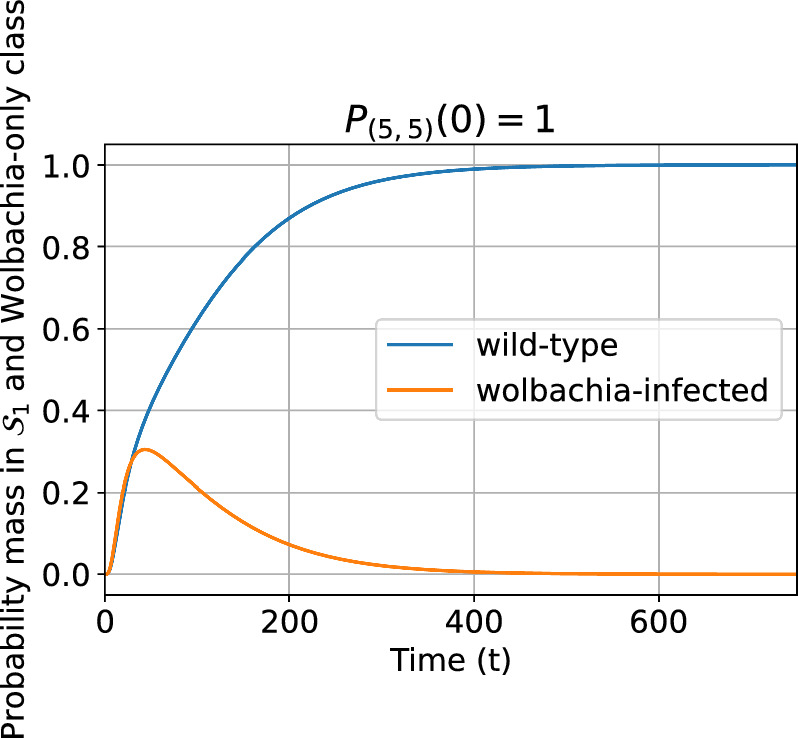
Dynamics of the probability distribution for the wildtype-only class and Wolbachia-only classes conditioned on non-extinction when vertical transmission is imperfect. Note that the Wolbachia-only class is not a communicating class here. The class probability is the sum of the probabilities for all states in that class. For the model with households of up to 30 female mosquitoes, $v=0.9$, $1-\phi =0.15$, larval density function is (6). Initially $P_{(5,5)}(0)=1$.

**Fig. 26:**
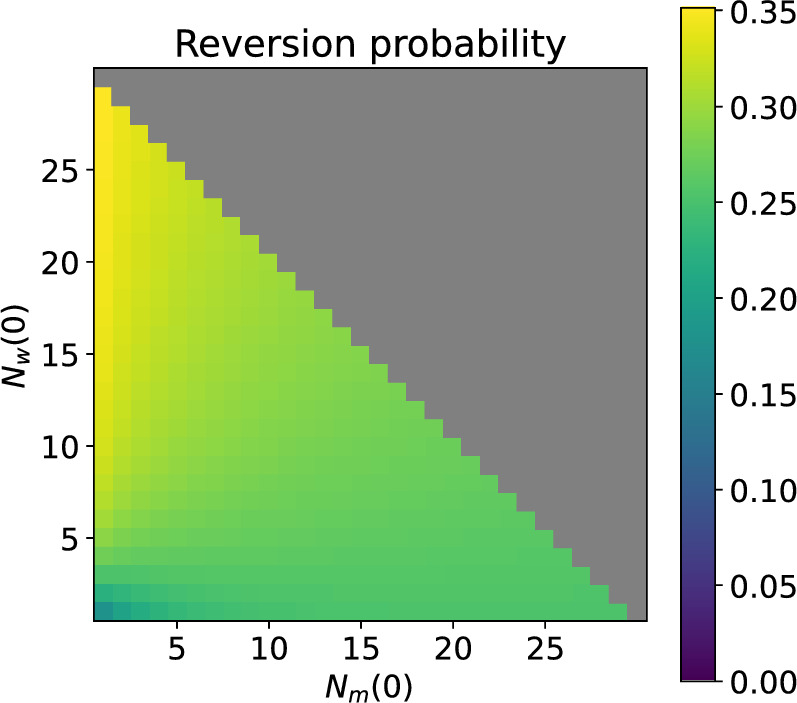
Probability of reversion from the Wolbachia-only class to the mixed class when the Wolbachia-only class is reached from an initial mixed state $(N_m(0), N_w(0))$. For the model with households of up to 30 mosquitoes, $v=0.9$, $1-\phi =0.15$, larval density function is (6).

**Fig. 27:**
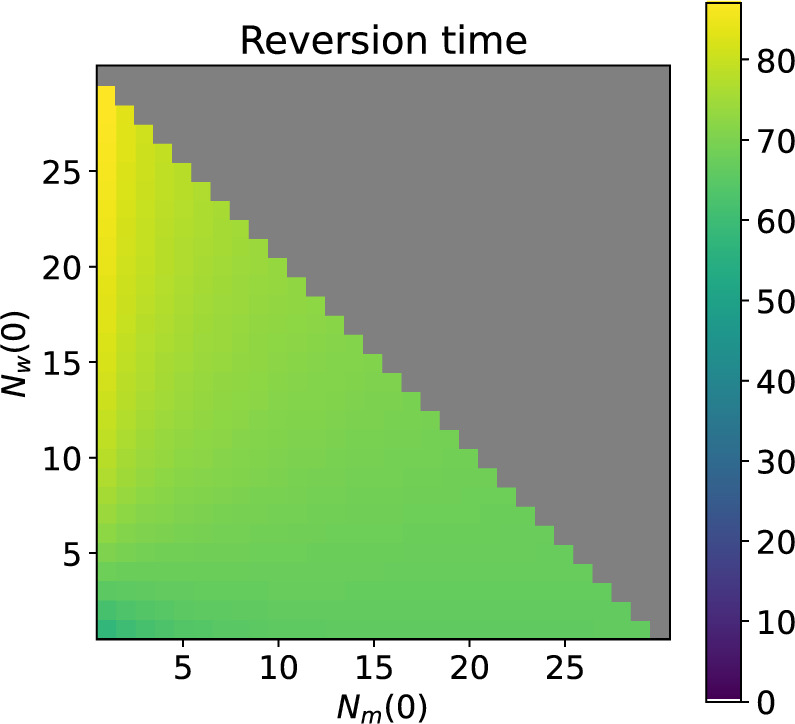
Expected time until reversion (in days) from the Wolbachia-only class to the mixed class (conditional on the event that reversion occurs) when the Wolbachia-only class is reached from an initial mixed state $(N_m(0), N_w(0))$. For the model with households of up to 30 mosquitoes, $v=0.9$, $1-\phi =0.15$, larval density function is (6).

**Fig. 28:**
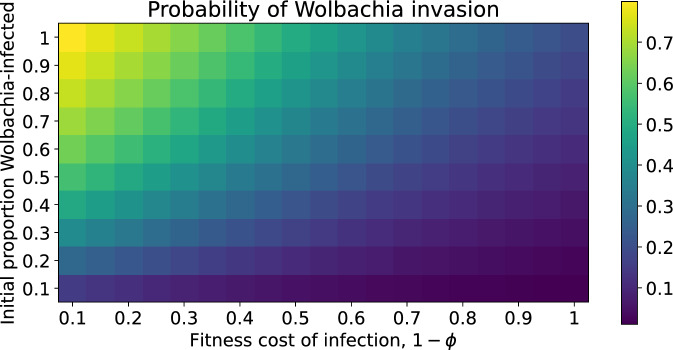
Probability of successful Wolbachia invasion of a household depending on the initial (female) Wolbachia-infected proportion $N_w/ (N_0+N_w)$ and fitness cost of infection $1- \phi$. At first the household is composed of $N_0=10$ female wildtype mosquitoes and between 1 and 10 additional Wolbachia-infected females are introduced so the total household population is between 11 and 20. Households can have up to 30 female mosquitoes and $v=1$ and the larval density function is (5).

**Fig. 29:**
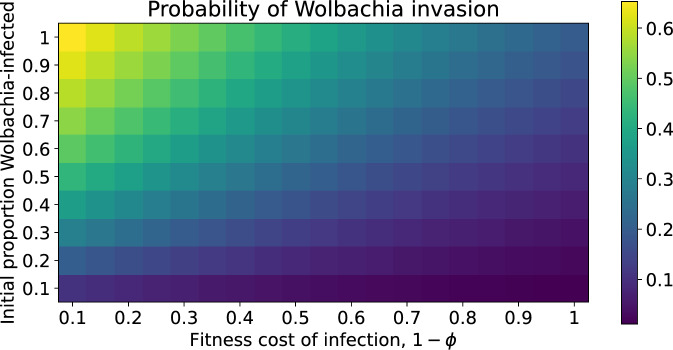
Probability of successful Wolbachia invasion of a household depending on the initial (female) Wolbachia-infected proportion $N_w/ (N_0+N_w)$ and fitness cost of infection $1- \phi$. At first the household is composed of $N_0=10$ female wildtype mosquitoes and between 1 and 10 additional Wolbachia-infected females are introduced so the total household population is between 11 and 20. Households can have up to 30 female mosquitoes and $v=0.9$ and the larval density function is (5).

**Fig. 30:**
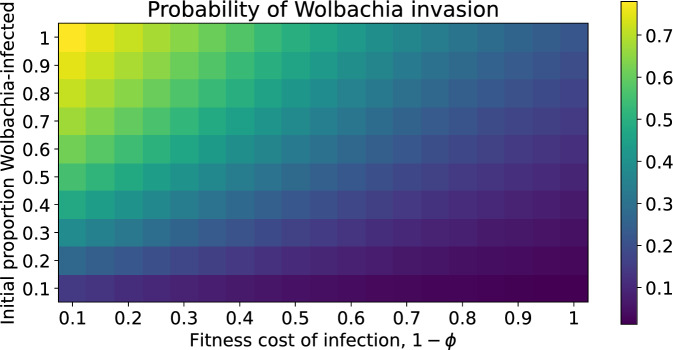
Probability of successful Wolbachia invasion of a household depending on the initial (female) Wolbachia-infected proportion $N_w/ (N_0+N_w)$ and fitness cost of infection $1- \phi$. At first the household is composed of $N_0=10$ female wildtype osquitoes and between 1 and 10 additional Wolbachia-infected females are introduced so the total household population is between 11 and 20. Households can have up to 30 female mosquitoes and $v=1$ and the larval density function is (6).

**Fig. 31:**
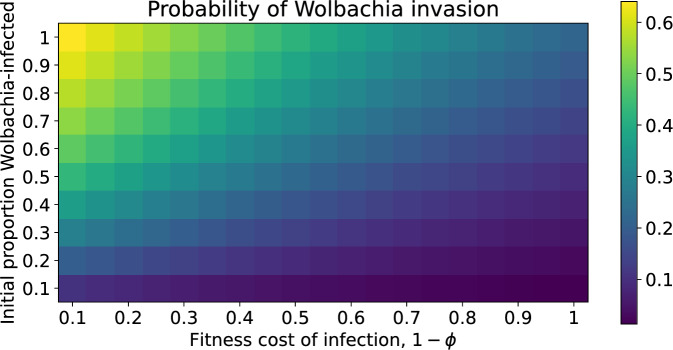
Probability of successful Wolbachia invasion of a household depending on the initial (female) Wolbachia-infected proportion $N_w/ (N_0+N_w)$ and fitness cost of infection $1- \phi$. At first the household is composed of $N_0=10$ female wildtype mosquitoes and between 1 and 10 additional Wolbachia-infected females are introduced so the total household population is between 11 and 20. Households can have up to 30 female mosquitoes and $v=0.9$ and the larval density function is (6).

**Fig. 32:**
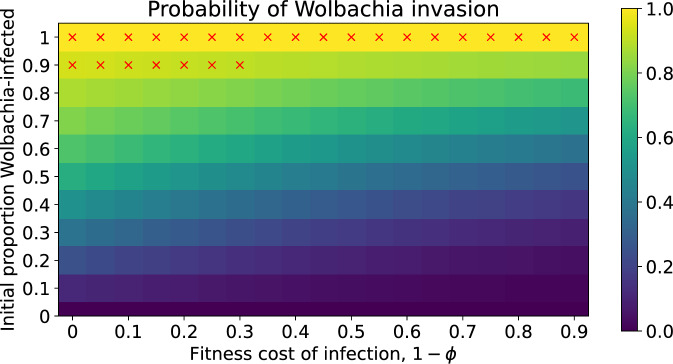
Probability of successful Wolbachia invasion of a household depending on the initial Wolbachia-infected proportion $N_w/N_0$, fitness cost of infection $1-\phi$, $v=0.9$ with larval density function (5). The initial household size is fixed at $N_0=10$ female mosquitoes and the initial infected proportion is determined by $N_w(0)$ with $N_m(0) + N_w(0) = N_0$. Households can have up to 30 female mosquitoes. The red crosses indicate where the probability of successful Wolbachia invasion is greater than or equal to 0.9.

**Fig. 33:**
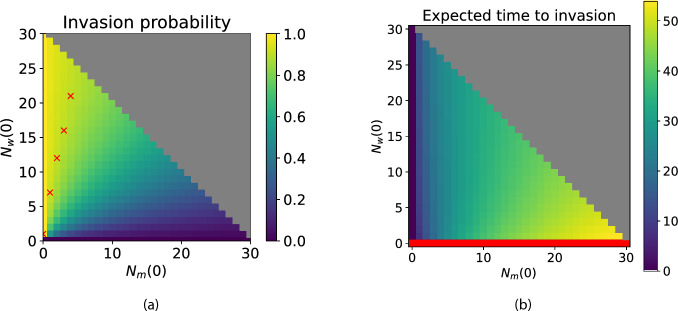
(a) Probability of entering the Wolbachia-infected class when the initial state is $(N_m(0), N_w(0))$. Red crosses denote, for different initial wildtype mosquito numbers, the minimum number of Wolbachia-infected mosquitoes required for successful invasion with probability 0.9 or higher. Note that, if there are more than 4 wildtype female mosquitoes present, the number of Wolbachia-infected mosquitoes required for a successful invasion with probability 0.9 or higher is outside of the state space. (b) Expected time in days to enter the Wolbachia-infected class, conditional on this event occurring, when the initial state is $(N_m(0), N_w(0))$. The red bar indicates initial conditions where only wildtype mosquitoes are present and so the Wolbachia-infected class cannot be reached. In both diagrams the maximum number of female mosquitoes in a household is 30, the initial state probability distribution is $P_{(N_m(0),N_w(0))}(0)=1$, parameters are as in Table 1, $1-\phi =0.15$, $v=0.9$, and the larval density function is (5). The top right quadrant shaded grey is not an admissible region of the state space.

**Fig. 35:**
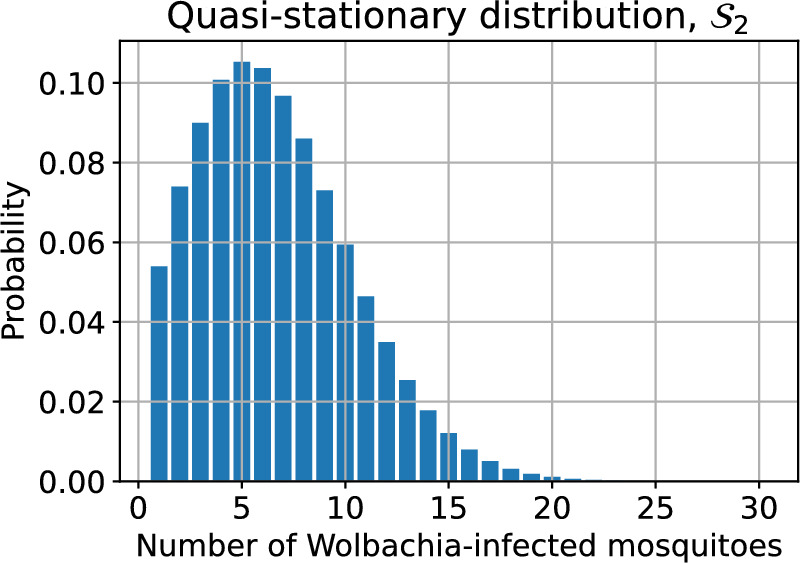
QSD for the Wolbachia-only class for the model with households of up to 30 female mosquitoes, perfect vertical transmission ($v=1$), $1-\phi =0.15$ and larval density function 5. Any states not shown have probability close to 0.

## Quasi-stationary distribution

### QSD from a reducible transition matrix

The QSD is straightforward to determine when the transition matrix $\boldsymbol{Q}$ is irreducible. But when $\boldsymbol{Q}$ is reducible, finding the QSD is more challenging. This is the case with our model. In order to find the QSD when the transition matrix is reducible, the state space must first be partitioned into its communicating classes. Here we follow the approach in Doorn and Pollett (2013) and write the states in the concise form of single letters. For two states *i* and *j*, we write $i \rightarrow j$ if the probability of reaching state *j* from state *i* via a series of state transitions is non-zero. Two states *i* and *j* are said to communicate, $i \leftrightarrow j$, if $i \rightarrow j$ and $j\rightarrow i$. The relation $\leftrightarrow$ is an equivalence relation on $\mathcal {S}$ that partitions $\mathcal {S}$ into subsets known as communicating classes. If $\boldsymbol{Q}$ is irreducible then the state space is composed of a single communicating class.

Suppose $\boldsymbol{Q}$ is reducible and $\mathcal {S}$ is composed of $L > 1$ communicating classes denoted $\mathcal {S}_{1},\mathcal {S}_{2}, \ldots , \mathcal {S}_L$. Let $\boldsymbol{Q}_k$ be the submatrix of $\boldsymbol{Q}$ composed of the transition rates between the states in $\mathcal {S}_{k}$. A class $\mathcal {S}_{i}$ is accessible from a class $\mathcal {S}_j$, written $\mathcal {S}_{i} \prec \mathcal {S}_j$, if starting from any state in $\mathcal {S}_j$, we are able to reach any state in $\mathcal {S}_{i}$ via some sequence of state transitions. Since $\mathcal {S}_{i}$ and $\mathcal {S}_j$ are distinct communicating classes, the converse is not true; $\mathcal {S}_j$ cannot be reached from $\mathcal {S}_{i}$. We order the communicating classes in such a way that $\mathcal {S}_{i}\prec \mathcal {S}_{j}$ implies that $j > i$; such an ordering exists because there cannot exist *i*, *j*, *k* distinct with $\mathcal {S}_{i} \prec \mathcal {S}_{j} \prec \mathcal {S}_{k}$ and $\mathcal {S}_{i}\succ \mathcal {S}_{k}$, since $\mathcal {S}_{i}, \mathcal {S}_{j}, \mathcal {S}_{k}$ are distinct communicating classes. One might visualise this structure as a cascade — probability mass always moves through the communicating classes down the ordering. The lowest class in the ordering, $\mathcal {S}_{1}$, is termed the minimal class (Doorn and Pollett 2013). The states themselves must be labelled in such a way that $\boldsymbol{Q}$ is of lower-triangular block form and the sub-*Q* matrices, $\boldsymbol{Q}_k$, reside along the diagonal of $\boldsymbol{Q}$. If necessary, the states can be relabelled to achieve this. The matrices $\boldsymbol{Q}_k$ must be ordered as described above, with the matrix $\boldsymbol{Q}_1$ positioned in the top left hand corner of $\boldsymbol{Q}$. This is the transition matrix that corresponds to communicating class $\mathcal {S}_{1}$, which is accessible from at least one of the classes higher up the order e.g. $\mathcal {S}_{2}$, $\mathcal {S}_{3}$ etc.

The set of eigenvalues of $\boldsymbol{Q}$ is equal to the union of the sets of eigenvalues of the $\boldsymbol{Q}_k$ matrices. The minimal magnitude eigenvalues of each $\boldsymbol{Q}_k$, which we denote $-\alpha _k$, are all unique (within the set of eigenvalues of $\boldsymbol{Q}_k$), simple and negative. Hence, the minimal magnitude eigenvalue of $\boldsymbol{Q}$ is the smallest of the set of minimal magnitude eigenvalues of the $\boldsymbol{Q}_k$. That is, the minimal magnitude eigenvalue of $\boldsymbol{Q}$ is $-\alpha := -\min _k \alpha _k$ (Seneta 2006). The QSD is, as in the irreducible case, the left eigenvector of $\boldsymbol{Q}$ corresponding to $-\alpha$ normalised to sum to 1. If the algebraic multiplicity of $-\alpha$ is greater than one, then $\alpha = \alpha _k$ in at least two values of *k*. In this case, the QSD is given by the eigenvalue-eigenvector pair of $\boldsymbol{Q}$ corresponding to the minimal communicating class, as defined above.

### State distribution in the main text stochastic model, $v=1$

Figure 16 shows the quasi-stationary distribution for the system modelled in Sect. 5.1 in the main text.

## Alternative larval density dependence function

All results in the main text come from models that use the function *F*(*N*) for density dependent competition at the larval stage where

B1 $$\begin{aligned} F(N)={\left\{ \begin{array}{ll}\exp (-hN^k) & N < C,\\ 0 & N \ge C. \end{array}\right. } \end{aligned}$$

This function was first introduced in Dye (1984), and used in many subsequent studies including (Hughes and Britton 2013). A simpler alternative to *F*(*N*) is *G*(*N*) given by Qu et al. (2022)

B2 $$\begin{aligned} G(N) = {\left\{ \begin{array}{ll} 1-N/C & N < C,\\ 0 & N \ge C. \end{array}\right. } \end{aligned}$$

Here we reproduce our main results using *G*(*N*). We use the same parameter regime as in Table 1. As before, we determine the per capita birth rate *b* by fixing the wildtype-only steady state at $x^*=10$ and solving $bx^*G(x^*)=dx^*$. The shape of *G*(*N*) results in $b=0.18$, much lower than the $b=0.54$ obtained with *F*(*N*).

### Bistability in the mean-field model

Figure 17 shows the stability and basins of attraction of the steady states of the mean-field model (1)–(2). As before (Fig. 2) we observe a bistable region. However, the lower birth rate associated with *G*(*N*) means that the bistable region is much smaller as the Wolbachia-only steady state only exists when $1-\phi <0.33$.

Figure 18 shows the basins of attraction across the full set of feasible initial conditions at a parameter location in the bistable region. The alternative density dependence function *G*(*N*) does not significantly change the results (compare with Fig. 3).

### State distributions and invasion probabilities in the stochastic model, $v=1$

The larval density dependence function does not affect the structure of the communicating classes (Sect. 5). The overall minimal magnitude eigenvalue, and hence the QSD, is still associated with the wildtype-only class. Figure 19 shows the QSD (which has all of its mass in the wildtype-only class). The alternative density dependence function *G*(*N*) has little impact on the QSD (compare with Fig. 16) except that the tail to the right is slightly smaller due to the lower birth rate.

Figure 20 shows the probabilities over time of a household being in the wildtype-only and Wolbachia-only classes, conditioned on non-extinction and initial state probability distribution $P_{(5,5)}(0)=1$. The alternative density dependence function *G*(*N*) has little impact on the distribution (compare with Fig. 7) but the distribution converges slightly faster to the QSD. This observation is corroborated by the damping ratio.

Figure 21 shows how the fitness cost of infection $1-\phi$ affects the probability of successful Wolbachia invasion (i.e. reaching a Wolbachia-only state) in a household containing a total of $N_0=10$ female mosquitoes, of which a proportion $N_w(0)/N_0$ are Wolbachia-infected. The alternative density dependence function *G*(*N*) has little impact on the correspondence between the deterministic bistability and the stochastic invasion probability (compare with Fig. 8). As such, *G*(*N*) tends to shift regions with high probability of reaching the Wolbachia-only state to lower values of $1-\phi$. There is little change between Figs. 9a and 22a. However, the expected time until Wolbachia invasion increases overall (compare Figs. 9b and 22b) due to the decrease in birth rate.

Figure 23 shows the individual state entropies, a measure of the uncertainty in the next state to be visited. The alternative density dependence function *G*(*N*) has little impact on the state entropies, except close to the boundary $N_m+N_w = C$, where they are markedly reduced (compare with Fig. 10). This reduction is due to a much stronger larval density effect close to the maximum population size *C* (see Fig. 1) which reduces the probability of a birth occurring in states close to the boundary.

Figure 24 shows the expected time until extinction for a Wolbachia-only household. The alternative density dependence function *G*(*N*) reduces the time to extinction (compare with Fig. 11). This reduction is a consequence of the lower birth rate.

### State distributions and invasion probabilities in the stochastic model with reversion, $v=0.9$

We next present results for the model with households of up to 30 female mosquitoes with parameters comparable to the mean-field model (1)–(2) where vertical transmission is imperfect, $v=0.9$. As above, we use the larval density dependence function (6) and set the fitness cost of Wolbachia infection to $1-\phi =0.15$ unless otherwise stated. The communicating class structure is the same as in Sect. 6. The QSD is once again identical to the QSD in the previous Sect. B.2 (see Sect. 6.2), so is omitted.

The probability distribution of the household states over time (conditioned on non-extinction and $P_0(5,5)=1$; Figure 25) is similar to that of Fig. 12 but converges slightly faster to the QSD.

Repeating the sensitivity analysis we carried out in Sect. 6 with the alternative larval density dependence function *G*(*N*), we obtain similar results (Fig. 26 and Fig. 27), although the reversion probability is slightly lower. The average reversion probability conditioned on initially reaching the Wolbachia-only state from an initial mixed state (*m*, *w*) for $0<m,w\le 30$ is 0.29 and the reversion probability assuming we enter the Wolbachia-only class via (5, 5) is 0.27. These lower values are due to the decrease in the wildtype birth rate. The expected time until reversion (conditional on the event that reversion occurs) also decreases. The average conditional expected time until reversion is 71 days and the conditional expected time to reversion from a critical mixed state (5, 5) is 68 days.

## Invasion probability with population size unconserved

In this section, we show plots for the probability of Wolbachia invasion into a wildtype-only mosquito household currently at its deterministic steady state value, $N_m^* = 10$. We vary the number of Wolbachia-infected mosquitoes released as a proportion of the initial wildtype household size $N_m(0)$, and add these to the initial wildtype population rather than replacing the corresponding number of wildtype mosquitoes. We vary the fitness cost of infection $1-\phi$ between 0.1 and 1. Note that here we are allowing the total number of mosquitoes in the household to be pushed above the steady state value. The plots include both larval density dependence functions (5) (see Figs. 28 and 29) and (6) (see Figs. 30 and 31) and the cases where $v=1$ and $v=0.9$.

## Wolbachia invasion results, $v=0.9$

In this section we present results on the probability of Wolbachia invasion and the expected time until invasion consistent with those from Sect. 5, except $v=0.9$ so that reversion is possible. Here we use the original larval density function *F*(*N*) from the main text (5). The results are similar to when $v=1$. However, observe the increase in the proportion of Wolbachia-infected mosquitoes required in a household of size 10 to achieve a successful invasion with probability 0.9 in Fig. 32 (compare with Fig. 8).

In Fig. 33a the minimum number of Wolbachia-infected mosquitoes required for the probability of invasion to be at least 0.9 has dramatically increased (as well as all the invasion probabilities across the initial condition space) compared to Fig. 9. This is because the Wolbachia-infected birth rate is now leaky. The expected time until Wolbachia invasion in Fig. 33b remains comparable to Fig. 9b. The state entropies in Fig. 34 are comparable to those in Fig. 10.

## Calculating the time until Wolbachia extinction

Here, we use the same Markov chain theory as in Sect. 3.4 (Norris 1997; Gómez-Corral and López García 2012) to determine the expected time until extinction of a Wolbachia-only population when $v=1$ so that reversion cannot occur. Recall $\boldsymbol{Q}_2$ is the sub-*Q* matrix corresponding to the Wolbachia-only class and the probability of eventually entering the absorbing state (0, 0) from any state in $\mathcal {S}$ is 1. The quantity $\textbf{q}(0,0)$ is the vector of transition rates from each state in $\mathcal {S}_{2}$ to the absorbing state. The expected time until extinction of a Wolbachia-only household in state $(0,w')$ is the solution of the linear system,

E3 $$\begin{aligned} \sum _{(0,l)\in \{(0,0)\}\cup \mathcal {S}_{2}} q_{(0,w'),(0,l)}\tau _{(0,l)}(0,0)=-1\;\;\; {\text {for}}\;\;\; (0,w')\in \mathcal {S}_{2}, \end{aligned}$$

where $q_{(0,w'),(0,l)}$ is the transition rate from state $(0,w')$ to state $(0,l)\in \{(0,0)\}\cup \mathcal {S}_{2}$ and $\tau _{(0,l)}(0,0)$ is the expected time for the household to enter the absorbing state (0, 0) from state (0, *l*).

## The Wolbachia-only QSD

The QSD for the Wolbachia-only class is found by setting $v=1$ under the 30 mosquito CTMC model. This ensures that the wildtype-only class is not accessible via the Wolbachia-only class. Then the QSD is the left eigenvector, normalised to sum to 1, of the minimal magnitude eigenvalue of $\boldsymbol{Q}_2$, the transition matrix for the for the Wolbachia-only class. The Wolbachia-only QSD is shown in Fig. 35.

## Deriving the damping ratio for a CTMC model

We can omit the absorbing state (0, 0) and its corresponding eigenvalue from Eq. (9) in order to consider just the transient subsystem (Mubayi et al. 2019). Let $-\lambda _1=-\alpha$, be the minimal magnitude eigenvalue and let $-\lambda _2$ be the eigenvalue of second smallest magnitude. Consequently, we can write

G4 $$\begin{aligned} \boldsymbol{P}(t)=\sum _{i=1}^{s-1}c_i e^{-\lambda _it}\boldsymbol{l}_i, \end{aligned}$$

and if we divide Eq. (9) by $\exp {(-\lambda _1 t)}$ we get

G5 $$\begin{aligned} \frac{\boldsymbol{P}(t)}{e^{-\lambda _1 t}} = c_1\boldsymbol{l}_1 + c_2\left( \frac{e^{-\lambda _2}}{e^{-\lambda _1}}\right) ^t \boldsymbol{l}_2 + \ldots , \end{aligned}$$

and taking the limit,

G6 $$\begin{aligned} \lim _{t \rightarrow \infty } \frac{\boldsymbol{P}(t)}{e^{-\lambda _1 t}}= c_1\boldsymbol{l}_1. \end{aligned}$$

Therefore, it follows that Caswell (2000)

G7 $$\begin{aligned} \lim _{t \rightarrow \infty } \left( \frac{\boldsymbol{P}(t)}{e^{-\lambda _1 t}}-c_1\boldsymbol{l}_1 - c_2\rho ^{-t}\boldsymbol{l}_2\right) =0, \end{aligned}$$

where the damping ratio $\rho$ is defined as

G8 $$\begin{aligned} \rho =\exp ((-\lambda _1)-(-\lambda _2))=\exp (\lambda _2-\lambda _1). \end{aligned}$$

## Deriving the expected time until Wolbachia invasion

Here, we derive the expected time $\tau ^*_{(k,l)}$ starting from state $(k,l) \in \mathcal {S}_{3}\cup \mathcal {S}_{2}$ until communicating class $\mathcal {S}_{2}$ is hit, given that $\mathcal {S}_{1}$ is not entered first. Adapted from Gómez-Corral and López García (2012), $\tau ^*_{(k,l)}$ is the minimal solution of (13) in Sect. 3.4.

To show this here, we relabel the states with a single letter for ease of notation. For $i\in \mathcal {S}_{3}\cup \mathcal {S}_{2}$, we can write the expected time to hit communicating class $\mathcal {S}_{2}$ given that the starting state is *i* and that $\mathcal {S}_{1}$ is not reached first,

H9 $$\begin{aligned} \tau _i^*=\mathbb {E}[T|X(0)=i,X(T)\in \mathcal {S}_{2}], \end{aligned}$$

where $\mathcal {X}:=\{X(t), t\ge 0\}$ is the Markov chain and $T=\inf \{t\ge 0:X(t)\in \mathcal {S}_{1}\cup \mathcal {S}_{2}\}$ is the first time we leave the mixed class. Now, by the conditional expectation formula,

H10 $$\begin{aligned} \tau _i(w)=\frac{\mathbb {E}_i\left[ T\mathbbm {1}_{\{X(T)\in \mathcal {S}_{2}\}}\right] }{\mathbb {P}_i(X(T)\in \mathcal {S}_{2})}, \end{aligned}$$

where we use the standard notation $\mathbb {P}_i(\cdot )=\mathbb {P}(\cdot | X(0)=i)$ and $\mathbb {E}_i(\cdot )=\mathbb {E}[\cdot |X(0)=i]$. Let $a_i^*=\mathbb {P}_i(X(T)\in \mathcal {S}_{2})$; then for $i\in \mathcal {S}_{3}$, letting $J_1$ denote the first jump time of $\mathcal {X}$ and letting $\pi _{ij}=\mathbb {P}(X(J_1)=j|X(0)=i)$ denote an entry of the jump matrix associated with $\boldsymbol{Q}$, we can write

H11 $$\begin{aligned} \begin{aligned} \tau _i^*a_i^*&= \mathbb {E}_i\left[ T\mathbbm {1}_{\{X(T)\in \mathcal {S}_{2}\}}\right] \\&=\mathbb {E}_i\left[ (J_1+ (T-J_1))\mathbbm {1}_{\{X(T)\in \mathcal {S}_{2}\}}\right] \\&=\mathbb {E}_i\left[ J_1\mathbbm {1}_{\{X(T)\in \mathcal {S}_{2}\}}\right] +\sum _{j\ne i, \;j\in \mathcal {S}_{3}\cup \mathcal {S}_{2}} \pi _{ij}\mathbb {E}_j\left[ T\mathbbm {1}_{\{X(T)\in \mathcal {S}_{2}\}}\right] . \end{aligned} \end{aligned}$$

Where the last line follows by the strong Markov property at time $J_1$. Further, since $J_1$ and $\{X(t+J_1),t\ge 0\}$ are independent and since $\pi _{ij}=q_{ij}/q_i$ by the jump matrix definition (Norris 1997), we can write

H12 $$\begin{aligned} \begin{aligned} \tau _i^*a_i^*&=\mathbb {E}_i[J_1]\mathbb {E}_i\left[ \mathbbm {1}_{\{X(T)\in \mathcal {S}_{2}\}}\right] +\sum _{j\ne i,\;j\in \mathcal {S}_{3}\cup \mathcal {S}_{2}}\frac{q_{ij}}{q_i}\tau _j^*a_j^*\\&=q^{-1}_i\mathbb {P}_i(X(T)\in \mathcal {S}_{2})+\sum _{j\ne i,\;j\in \mathcal {S}_{3}\cup \mathcal {S}_{2}} \frac{q_{ij}}{q_i}\tau _j^*a_j^*\\&=q^{-1}_ia_i^* + \sum _{j\ne i,\;j\in \mathcal {S}_{3}\cup \mathcal {S}_{2}} \frac{q_{ij}}{q_i}\tau _j^*a_j^*, \end{aligned} \end{aligned}$$

where the second line follows since, conditional on $X(0)=i$, $J_1$ is exponentially distributed with rate $q_i$. Therefore,

H13 $$\begin{aligned} \begin{aligned}&\tau _i^*a_i^*q_i=a_i^*+\sum _{j\ne i,\;j\in \mathcal {S}_{3}\cup \mathcal {S}_{2}} q_{ij}\tau _j^*a_j^*\\&\Rightarrow \sum _{j\in \mathcal {S}_{3}\cup \mathcal {S}_{2}} q_{ij}\tau _j^*a_j^*=-a_i^*, \end{aligned} \end{aligned}$$

since $q_i=-q_{ii}$. This yields the required result (13) in the compact notation.

## Eigenvalue interpretation justification

### Claim 1: Time for probability mass in class to become negligible

We claimed that the time for the probability mass in class $\mathcal {S}_{k}$ in our system to become negligible is governed by the minimal magnitude eigenvalue of $\boldsymbol{Q}_k$. We will now prove this.

Let $\boldsymbol{Q}_k$ denote the matrix $\boldsymbol{Q}$ restricted to $\mathcal {S}_{k}$ and let $a_k=\max _{i\in \mathcal {S}_{k}}(-\boldsymbol{Q}_{ii})>0$, the maximal magnitude of the diagonal entries of $\boldsymbol{Q}_k$. Since $\mathcal {S}_{k}$ is a communicating class, for all $i,j\in \mathcal {S}_{k}$, there exist an $n \in \mathbb {N}$ and $i=i_0,i_1,\ldots ,i_n=j\in \mathcal {S}_{k}$ such that $(\boldsymbol{Q}_k)_{i_r,i_r+1}>0$ for all $r \in \{0,\ldots ,n-1\}$. Therefore $\boldsymbol{Q}_k+a_kI$ is a non-negative irreducible matrix. Hence we can apply the Perron-Frobenius Theorem to $\boldsymbol{Q}_k + a_kI$. This tells us that there exists a unique left eigenvector $\boldsymbol{l}$ of the matrix $\boldsymbol{Q}_k + a_kI$ such that each entry of $\boldsymbol{l}$ is positive, and a unique right eigenvector $\boldsymbol{u}$ of the matrix $\boldsymbol{Q}_k + a_kI$ such that each entry of $\boldsymbol{u}$ is positive, both with the same positive eigenvalue $b_k$. The eigenvalue $b_k$ is the largest eigenvalue of $\boldsymbol{Q}_k + a_kI$, and is simple. Letting $-\alpha _k=b_k-a_k$, we have that $\boldsymbol{l}\boldsymbol{Q}_k=-\alpha _k\boldsymbol{l}$ and $\boldsymbol{Q}_k\boldsymbol{u}=-\alpha _k\boldsymbol{u}$, and $-\alpha _k$ is the largest eigenvalue of $\boldsymbol{Q}_k$ and is simple. Therefore, there exists an invertible matrix $\boldsymbol{A}_k$ such that $\boldsymbol{Q}_k=\boldsymbol{A}_k\boldsymbol{\Lambda }_k\boldsymbol{A}^{-1}_k$, where

I14 $$\begin{aligned} \boldsymbol{\Lambda }_k=\begin{pmatrix} -\alpha _k & \boldsymbol{0}_{1\times n} \\ \boldsymbol{0}_{n\times 1} & \boldsymbol{\Lambda }'_k \\ \end{pmatrix}, \end{aligned}$$

$n=|\mathcal {S}_{k}|-1$ and $\boldsymbol{0}_{n\times 1}=\boldsymbol{0}_{1\times n}^{\top }$ is a column vector of zeros and $\boldsymbol{\Lambda }'_k$ is a Jordan normal matrix with diagonal entries strictly less than $-\alpha _k$. Since $(\boldsymbol{Q}_k\boldsymbol{A}_k)_{i1}=\sum _{j\in \mathcal {S}_{k}}(\boldsymbol{Q}_k)_{ij}(\boldsymbol{A}_k)_{j1}=(\boldsymbol{A}_k\boldsymbol{\Lambda }_k)_{i1}=-\alpha _k(\boldsymbol{A}_k)_{i1}$, it holds that $(\boldsymbol{A}_k)_{i1}=\tilde{c}\boldsymbol{u}_i$ for some constant $\tilde{c}$. Since $(\boldsymbol{A}^{-1}_k\boldsymbol{Q}_k)_{1i}=\sum _{j\in \mathcal {S}_{k}}(\boldsymbol{A}^{-1}_k)_{1j}(\boldsymbol{Q}_k)_{ji}=(\boldsymbol{\Lambda }_k\boldsymbol{A}^{-1}_k)_{1i}=-\alpha _k(\boldsymbol{A}^{-1}_k)_{1i}$, we have also that $(\boldsymbol{A}^{-1}_k)_{1i}=c\boldsymbol{l}_i$ for some constant *c*. Since $\sum _{i \in \mathcal {S}_{k}}(\boldsymbol{A}_k^{-1})_{1i}(\boldsymbol{A}_k)_{i1}=1$, we have $\sum _{i\in \mathcal {S}_{k}}c\tilde{c}\boldsymbol{l}_i\boldsymbol{u}_i=1$. Therefore we can assume that $\boldsymbol{u}$ and $\boldsymbol{l}$ are chosen in such a way that $(\boldsymbol{A}_k)_{i1}=\boldsymbol{u}_i$, $(\boldsymbol{A}_k^{-1})_{1i}=\boldsymbol{l}_i$ and $\boldsymbol{l}\cdot \boldsymbol{u}=1$.

For $i,j\in \mathcal {S}_{k}$ and $t\ge 0$, we have Norris (1997)

I15 $$\begin{aligned} \mathbb {P}(X(t)=j|X(0)=i)=(e^{t\boldsymbol{Q}})_{ij} = \sum _{m=0}^{\infty }\frac{1}{m!}t^m(\boldsymbol{Q}^m)_{ij}=\sum _{m=0}^{\infty }\frac{1}{m!}t^m(\boldsymbol{Q}^m_k)_{ij}, \end{aligned}$$

since, by the class structure of the Markov chain, for $n\in \mathbb {N}$ and $i=i_0,i_1,\ldots ,i_n=j\in \mathcal {S}$ with $i,j\in \mathcal {S}_{k}$ and $i_{n'}\notin \mathcal {S}_{k}$ for some $n'\in \{1,\ldots ,n-1\}$ we have $\boldsymbol{Q}_{i_0,i_1}\boldsymbol{Q}_{i_1,i_2}\ldots \boldsymbol{Q}_{i_{n-1},i_n}=0$. Therefore

I16 $$\begin{aligned} \begin{aligned} \mathbb {P}(X(t)=j|X(0)=i)&=\sum _{m=0}^{\infty }\frac{1}{m!}t^m\left( \boldsymbol{A}_k\boldsymbol{\Lambda }_k\boldsymbol{A}^{-1}_k\right) ^m_{ij}=\sum _{m=0}^{\infty }\frac{1}{m!}t^m\left( \boldsymbol{A}_k\boldsymbol{\Lambda }_k^m\boldsymbol{A}^{-1}_k\right) _{ij}\\&=\left( \boldsymbol{A}_k\left( \sum _{m=0}^{\infty }\frac{1}{m!}t^m\boldsymbol{\Lambda }^m_k\right) \boldsymbol{A}^{-1}_k\right) _{ij}. \end{aligned} \end{aligned}$$

By (I14), we also have that

I17 $$\begin{aligned} \sum _{m=0}^{\infty }\frac{1}{m!}t^m\boldsymbol{\Lambda }^m_k=\begin{pmatrix} e^{-\alpha _kt} & \boldsymbol{0}_{1\times n} \\ \boldsymbol{0}_{n \times 1} & \boldsymbol{B}_k(t) \end{pmatrix}, \end{aligned}$$

where the entries of $\boldsymbol{B}_k(t)$ are all at most $t^{|\mathcal {S}_{k}|-2}e^{-\alpha '_k t}$, where $-\alpha '_k<-\alpha _k$ is the second largest eigenvalue of $\boldsymbol{Q}_k$. So substituting in (I16), it holds that as $t \rightarrow \infty$

I18 $$\begin{aligned} \begin{aligned} \mathbb {P}(X(t)=j|X(0)=i)&=(\boldsymbol{A}_k)_{i1}e^{-\alpha _kt}\left( \boldsymbol{A}^{-1}_k\right) _{1j} +O\left( t^{|\mathcal {S}_{k}|-2}e^{-\alpha '_kt}\right) \\&=e^{-\alpha _kt}\boldsymbol{u}_i\boldsymbol{l}_j+O\left( t^{|\mathcal {S}_{k}|-2}e^{-\alpha '_kt}\right) , \end{aligned} \end{aligned}$$

where eigenvectors $\boldsymbol{l}$ and $\boldsymbol{u}$ are both positive and satisfy $\boldsymbol{l}\boldsymbol{Q}_k=-\alpha _k\boldsymbol{l}$, $\boldsymbol{Q}_k\boldsymbol{u}=-\alpha _k\boldsymbol{u}$ and $\boldsymbol{l} \cdot \boldsymbol{u}=1$, and $-\alpha _k$ is the largest eigenvalue of $\boldsymbol{Q}_k$. This is the required result.

### Claim 2: Quasi-stationary distribution with a class

Secondly, in Sect. 3.2 we claimed that the probability dynamics in our system in $\mathcal {S}_{2}$ will enter their own kind of quasi-stationary distribution and that the same holds for $\mathcal {S}_{1}$ (which is trivial since the QSD of the full system only holds positive probability mass in $\mathcal {S}_{1}$). We prove this claim next below.

Fix $k\in \{1,2\}$ and $j \in \mathcal {S}_{k}$, then the probability that the Markov chain is at state *j* at time *t*, given that it started at state $i\in \mathcal {S}_{3}$ and given that it is in $\mathcal {S}_{k}$ at time *t* is

I19 $$\begin{aligned} \mathbb {P}(X(t)=j|X(t)\in \mathcal {S}_{k},X(0)=i)=\frac{\mathbb {P}(X(t)=j|X(0)=i)}{\mathbb {P}(X(t)\in \mathcal {S}_{k}|X(0)=i)}. \end{aligned}$$

Our strategy will be to show that given $X(0)=i$, the events $\{X(t)=j\}$ and $\{X(t)\in \mathcal {S}_{k}\}$ are very unlikely to occur unless the Markov chain is already in class $\mathcal {S}_{k}$ at an earlier time *ct*, for suitable $c<1$. Then between the times *ct* and *t*, the Markov chain can get close to the QSD in $\mathcal {S}_{k}$.

Recall from Sect. 5.1 that $\alpha _3>\alpha _k$ (see first row of Table 4). So we can take $c<1$ sufficiently large that $\alpha _3c>\alpha _k$. Then it holds that

I20 $$\begin{aligned} \begin{aligned} \mathbb {P}(X(t)=j|X(0)=i)&=\mathbb {P}(X(t)=j, X(ct)\in \mathcal {S}_{k}|X(0)=i) \\&+ \mathbb {P}(X(t)=j,X(ct)\in \mathcal {S}_{3}|X(0)=i). \end{aligned} \end{aligned}$$

That is, the probability the Markov chain is at state *j* at time *t* (given it started at state *i*) can be partitioned into the sum of the probabilities that the Markov chain has already entered $\mathcal {S}_{k}$ at time *ct* or that the Markov chain is still in $\mathcal {S}_{3}$ at time *ct*. Clearly,

I21 $$\begin{aligned} \begin{aligned} \mathbb {P}(X(t)=j,X(ct)\in \mathcal {S}_{3}|X(0)=i)&\le \mathbb {P}(X(ct)\in \mathcal {S}_{3}|X(0)=i)\\&=\sum _{j'\in \mathcal {S}_{3}} \mathbb {P}(X(ct)=j'|X(0)=i)=O\left( e^{-\alpha _3 ct}\right) , \end{aligned} \end{aligned}$$

where the last line follows by (I18) because $\mathbb {P}(X(ct)=j'|X(0)=i)=O\left( e^{-\alpha _3ct}\right)$ for each $j'\in \mathcal {S}_{3}$. For the first term on the right hand side of (I20), since if $X(1)\in \mathcal {S}_{k}$ and $X(t)\in \mathcal {S}_{k}$ then $X(ct)\in \mathcal {S}_{k}$, we can fix $j_0\in \mathcal {S}_{k}$ and write

I22 $$\begin{aligned} \begin{aligned} \mathbb {P}(X(t)=j,X(ct)&\in \mathcal {S}_{k}|X(0)=i)\ge \mathbb {P}(X(t)=j,X(1)=j_0|X(0)=i)\\&=\mathbb {P}(X(t-1)=j|X(0)=j_0)\mathbb {P}(X(1)=j_0|X(0)=i), \end{aligned} \end{aligned}$$

where the last line follows by the Markov property at time 1. By (I18), and since $j,j_0 \in \mathcal {S}_{k}$, we have $\mathbb {P}(X(t-1)=j|X(0)=j_0) \gtrsim e^{-\alpha _k(t-1)}$ as $t\rightarrow \infty$.

Since $\mathbb {P}(X(1)=j_0|X(0)=i)>0$ because $i \in \mathcal {S}_{3}$ and $j_0\in \mathcal {S}_{k}$ and so $i\rightarrow j_0$, it follows that $\mathbb {P}(X(t)=j,X(ct)\in \mathcal {S}_{k}|X(0)=i) \gtrsim e^{-\alpha _kt}$ as $t\rightarrow \infty$. Therefore,

I23 $$\begin{aligned} \begin{aligned} \frac{\mathbb {P}\left( X(t)=j,X(ct)\in \mathcal {S}_{3}|X(0)=i\right) }{\mathbb {P}\left( X(t)=j,X(ct)\in \mathcal {S}_{k}|X(0)=i\right) }&=O\left( e^{-\alpha _3ct+\alpha _kt}\right) \\&=O\left( e^{-c't}\right) , \end{aligned} \end{aligned}$$

where $c'=\alpha _3c-\alpha _k>0$ by our choice of *c*. By (I20) and (I23) we now have

I24 $$\begin{aligned} \begin{aligned} \mathbb {P}(X(t)=j|X(0)=i)=\left( 1+\epsilon _t^{(j)}\right) \mathbb {P}\left( X(t)=j,X(ct)\in \mathcal {S}_{k}|X(0)=i\right) , \end{aligned} \end{aligned}$$

where $\epsilon _t^{(j)}=O\left( e^{-c't}\right)$ as $t \rightarrow \infty$. Since (I24) holds for each $j\in \mathcal {S}_{k}$, we can also write

I25 $$\begin{aligned} \begin{aligned} \mathbb {P}(X(t)\in \mathcal {S}_{k}|X(0)=i)&=\sum _{j'\in \mathcal {S}_{k}}\mathbb {P}(X(t)=j'|X(0)=i)\\&=\sum _{j'\in \mathcal {S}_{k}}\left( 1+\epsilon _t^{(j')}\right) \mathbb {P}(X(t)=j', X(ct)\in \mathcal {S}_{k}|X(0)=i)\\&\le \left( 1+\max _{j'\in \mathcal {S}_{k}}\epsilon _t^{(j')}\right) \sum _{j'\in \mathcal {S}_{k}}\mathbb {P}(X(t)=j', X(ct)\in \mathcal {S}_{k}|X(0)=i)\\&=\left( 1+\max _{j'\in \mathcal {S}_{k}}\epsilon _t^{(j')}\right) \mathbb {P}(X(t)\in \mathcal {S}_{k}, X(ct)\in \mathcal {S}_{k}|X(0)=i). \end{aligned} \end{aligned}$$

Since we also have $\mathbb {P}(X(t)\in \mathcal {S}_{k}|X(0)=i)\ge \mathbb {P}(X(t)\in \mathcal {S}_{k}, X(ct)\in \mathcal {S}_{k}|X(0)=i)$, it follows from (I25) that for some $\epsilon '_t\ge 0$ with $\epsilon '_t\le \max _{j'\in \mathcal {S}_{k}}\epsilon ^{(j')}_t$, we have

I26 $$\begin{aligned} \begin{aligned} \mathbb {P}(X(t)\in \mathcal {S}_{k}|X(0)=i)=\left( 1+\epsilon '_t\right) \mathbb {P}(X(t)\in \mathcal {S}_{k},X(ct)\in \mathcal {S}_{k}|X(0)=i). \end{aligned} \end{aligned}$$

Substituting in (I24) and (I26) into (I19), we now have

I27 $$\begin{aligned} \begin{aligned} \mathbb {P}(X(t)&=j|X(t)\in \mathcal {S}_{k},X(0)=i)\\&=\frac{\left( 1+\epsilon _t^{(j)}\right) \mathbb {P}(X(t)=j, X(ct)\in \mathcal {S}_{k}|X(0)=i)}{\left( 1+\epsilon '_t\right) \mathbb {P}(X(t)\in \mathcal {S}_{k},X(ct)\in \mathcal {S}_{k}|X(0)=i)}\\&=\frac{1+\epsilon _t^{(j)}}{1+\epsilon '_t}\frac{\sum _{j'\in \mathcal {S}_{k}}\mathbb {P}(X(t)=j,X(ct)=j'|X(0)=i)}{\sum _{j'\in \mathcal {S}_{k}}\mathbb {P}(X(t)\in \mathcal {S}_{k},X(ct)=j'|X(0)=i)}\\&=\frac{1+\epsilon _t^{(j)}}{1+\epsilon '_t}\frac{\sum _{j'\in \mathcal {S}_{k}}\mathbb {P}(X(ct)=j'|X(0)=i)\mathbb {P}(X((1-c)t)=j|X(0)=j')}{\sum _{j'\in \mathcal {S}_{k}}\mathbb {P}(X(ct)=j'|X(0)=i)\mathbb {P}(X((1-c)t)\in \mathcal {S}_{k}|X(0)=j')}, \end{aligned} \end{aligned}$$

where the last line follows by the Markov property at time *ct*. For $j'\in \mathcal {S}_{k}$, by (I18) we have

I28 $$\begin{aligned} \begin{aligned} \frac{\mathbb {P}(X((1-c)t)=j|X(0)=j')}{\mathbb {P}(X((1-c)t)\in \mathcal {S}_{k}|X(0)=j')}=\frac{\mathbb {P}(X((1-c)t)=j|X(0)=j')}{\sum _{j''\in \mathcal {S}_{k}}\mathbb {P}(X((1-c)t)=j''|X(0)=j')}\\ =\frac{e^{-\alpha _k(1-c)t}\boldsymbol{u}_{j'}^{(k)}\boldsymbol{l}_j^{(k)}+O\left( t^{|\mathcal {S}_{k}|-2}e^{-\alpha '_k(1-c)t}\right) }{\sum _{j''\in \mathcal {S}_{k}}\left( e^{-\alpha _k(1-c)t}\boldsymbol{u}_{j'}^{(k)}\boldsymbol{l}_{j''}^{(k)}\right) +O\left( t^{|\mathcal {S}_{k}|-2}e^{-\alpha '_k(1-c)t}\right) }, \end{aligned} \end{aligned}$$

where $\boldsymbol{l}^{(k)}$ and $\boldsymbol{u}^{(k)}$ are the positive eigenvectors of $\boldsymbol{Q}_k$ with $\boldsymbol{l}^{(k)}\boldsymbol{Q}_k=-\alpha _k\boldsymbol{l}^{(k)}$, $\boldsymbol{Q}_k\boldsymbol{u}^{(k)}=-\alpha _k\boldsymbol{u}^{(k)}$ and $\boldsymbol{l}^{(k)} \cdot \boldsymbol{u}^{(k)}=1$ and $-\alpha _k'<-\alpha _k$ is the second largest eigenvalue of $\boldsymbol{Q}_k$. Therefore, since the $\boldsymbol{u}^{(k)}_{j'}$ terms and $e^{-\alpha _k(1-c)t}$ terms cancel, we have

I29 $$\begin{aligned} \begin{aligned} \frac{\mathbb {P}(X((1-c)t)=j|X(0)=j')}{\mathbb {P}(X((1-c)t)\in \mathcal {S}_{k}|X(0)=j')}=\frac{\boldsymbol{l}_j^{(k)}+O\left( t^{|\mathcal {S}_{k}|-2}e^{-(\alpha '_k-\alpha _k)(1-c)t}\right) }{\sum _{j''\in \mathcal {S}_{k}}\boldsymbol{l}_{j''}^{(k)}+O\left( t^{|\mathcal {S}_{k}|-2}e^{-(\alpha '_k-\alpha _k)(1-c)t}\right) }. \end{aligned} \end{aligned}$$

Finally, substituting into (I27), we can write

I30 $$\begin{aligned} \begin{aligned} \mathbb {P}(X(t)=j|X(t)\in \mathcal {S}_{k},X(0)=i)&=\frac{1+\epsilon _t^{(j)}}{1+\epsilon '_t}\left( \frac{\boldsymbol{l}_j^{(k)}}{\sum _{j''\in \mathcal {S}_{k}}\boldsymbol{l}_{j''}^{(k)}}+O\left( t^{|\mathcal {S}_{k}|-2}e^{-(\alpha '_k-\alpha _k)(1-c)t}\right) \right) \\&=\frac{\boldsymbol{l}_j^{(k)}}{\sum _{j''\in \mathcal {S}_{k}}\boldsymbol{l}_{j''}^{(k)}}+O\left( e^{-c't}+t^{|\mathcal {S}_{k}|-2}e^{-(\alpha '_k-\alpha _k)(1-c)t}\right) . \end{aligned} \end{aligned}$$

This completes the proof of our claim.
